# Novel Hyperplastic Expansion of White Adipose Tissue Underlies the Metabolically Healthy Obese Phenotype of Male LFABP Null Mice

**DOI:** 10.3390/cells14110760

**Published:** 2025-05-22

**Authors:** Anastasia Diolintzi, Yinxiu Zhou, Angelina Fomina, Yifei Sun, Seema Husain, Labros S. Sidossis, Susan K. Fried, Judith Storch

**Affiliations:** 1 Department of Kinesiology and Health, Rutgers University, New Brunswick, NJ 08901, USA; lsidossis@kines.rutgers.edu; 2 Rutgers Center for Lipid Research, New Brunswick, NJ 08901, USA; 3 Department of Nutritional Sciences, Rutgers University, New Brunswick, NJ 08901, USAangelinafominav@gmail.com (A.F.); 4 The Mount Sinai Center for RNA Biology and Medicine, Icahn School of Medicine at Mount Sinai, New York, NY 10029, USA; yifei.sun@mssm.edu; 5 Department of Microbiology, Biochemistry and Molecular Genetics, Rutgers New Jersey Medical School, Newark, NJ 07103, USA; husainse@njms.rutgers.edu; 6 BioReference Laboratories Inc., Elmwood Park, NJ 07407, USA; 7 Diabetes, Obesity & Metabolism Institute (DOMI), Icahn School of Medicine at Mount Sinai, New York, NY 10029, USA; susan.fried@mssm.edu

**Keywords:** adipose tissue biology, adipogenesis, hyperplasia, adipose tissue remodeling, adipose tissue inflammation, cholesterol biosynthesis, diet-induced obesity, metabolically healthy obesity, metabolic health and disease, liver fatty acid-binding protein (LFABP; FABP1)

## Abstract

Obesity is an important risk factor for the development of metabolic syndrome disorders. We previously showed that the liver fatty acid-binding protein null mouse (*LFABP*^−/−^) becomes obese upon high-fat diet (HFD) feeding but remains metabolically healthy. Here, we find that the obese *LFABP*^−/−^ mouse increases subcutaneous adipose tissue (SAT) mass by markedly increasing the number rather than the size of adipocytes, as is typical with HFD. Indeed, while HFD-fed *LFABP*^−/−^ mice had almost double the fat mass of WT, SAT adipocyte size was >4-fold smaller and adipocyte number was 5-fold higher in the *LFABP*^−/−^. Transcriptomic analysis of SAT revealed that *Lfabp* deletion alters the expression of multiple pathways that modulate adipose expansion and function including cholesterol biosynthesis, adipogenesis, and extracellular matrix remodeling. LFABP is expressed in the liver and small intestine but not in adipose tissues; thus, its ablation may promote interorgan crosstalk that drives the hyperplastic expansion of metabolically beneficial SAT, contributing to the healthy obese phenotype of the *LFABP*^−/−^ mouse.

## 1. Introduction

The mammalian fatty acid-binding protein (FABP) family includes over 10 distinct members, each with a unique tissue distribution pattern. The FABPs are localized primarily to the cytosolic compartment of cells. Liver fatty acid-binding protein (LFABP; FABP1) is expressed in high abundance within both the liver and the proximal small intestine [1]. In addition to long-chain fatty acids (LCFAs), LFABP binds other lipid species including lysophospholipids, monoacylglycerols (MAGs), fatty acyl-CoAs, endocannabinoids (eCBs), bile acids (BAs), and prostaglandins [2]. When challenged with a high-fat diet (HFD), *LFABP* null (*LFABP*^−/−^) mice become substantially more obese compared to their wild-type (WT) counterparts, but they maintain a metabolically healthy obese (MHO) phenotype characterized by normoglycemia, normoinsulinemia, decreased hepatic steatosis and increased spontaneous physical activity [3]. Intriguingly, HF-fed *LFABP*^−/−^ mice, despite their greater adiposity, are also protected from an HFD-induced decline in exercise capacity, displaying an approximate doubling of running distance and time to exhaustion compared with WT mice [4]. Associated metabolic alterations include elevated plasma free fatty acid (FFA) levels post-exercise [4], which may be indicative of increased adipose tissue lipolysis to fuel the exercising muscle.

During overweight/obesity development, adipose tissue (AT) expands through a combination of adipocyte hypertrophy, i.e., an increase in the size of existing cells, and hyperplasia, i.e., the recruitment, proliferation, and differentiation of new adipocyte progenitor cells (APCs) in a process called adipogenesis [5,6,7]. The recruitment of inflammatory cells and vascular and extracellular matrix (ECM) remodeling occur as well, allowing sufficient tissue expansion, oxygen supply, and nutrient mobilization [8,9,10,11]. Hyperplastic AT growth is considered to be the preferred mechanism of expansion since it protects against metabolic disease by maintaining normal adipocyte size and function, with sufficient lipid storage capacity within AT [12,13]. An inability to recruit new adipocytes leads to an enlargement of the pre-existing adipocytes, which is thought to be associated with macrophage infiltration, inflamed and dysfunctional AT, ectopic lipid deposition in non-adipose tissues, as well as local and systemic insulin resistance (IR), all contributing to disease development [14]. In general, the mechanisms by which obesity drives hyperplastic instead of hypertrophic adipose tissue expansion remain poorly understood.

Excess energy storage is normally accomplished by subcutaneous white adipose tissue (sWAT); when its storage capacity is exceeded, excess calories accumulate in visceral WAT (vWAT) depots and ectopic sites such as the liver and muscle, causing lipotoxic insult [15,16]. Hence, homeostatic remodeling of AT expansion becomes dysfunctional in the context of hypertrophic obesity and sustained energy surplus, with increased adipocyte turnover, adipocyte IR, excess ECM deposition (i.e., fibrosis), reduced angiogenic remodeling, and infiltration of immune cells, thus shaping a proinflammatory and fibrotic milieu [8,11,17].

*LFABP* null mice show marked increases in subcutaneous and visceral fat mass relative to WT [3]; however, nothing is known regarding the tissue cellularity or the mechanism of expansion of these fat depots, nor is there information about brown adipose tissue (BAT) in the *LFABP*^−/−^ mouse. In the present study, we sought to characterize the effects of *Lfabp* ablation on AT during diet-induced obesity (DIO) development using histological, transcriptomic, and physiological analyses, to better understand the MHO phenotype of *LFABP*^−/−^ mice. The results demonstrate a highly unusual hyperplastic expansion of sWAT in the HFD-fed LFABP null mouse, suggesting that AT may be an important determinant of their MHO phenotype.

## 2. Materials and Methods

### 2.1. Experimental Design

Male mice on a C57Bl/6J background, as previously described [18,19], were separated into four groups: (a) WT fed a low-fat diet (LFD), (b) WT fed an HFD, (c) *LFABP*^−/−^ fed an LFD, and (d) *LFABP*^−/−^ fed an HFD. Mice were maintained on a 12 h light/dark cycle and had unrestricted access to rodent chow (Purina Laboratory Rodent Diet 5015, W.F. Fisher & Son, Inc., Somerville, NJ, USA). Mice were weaned onto a chow diet. At 8 weeks of age, WT and *LFABP*^−/−^ mice were housed 2–3 per cage and fed either a 45 kcal % fat diet high in saturated fat (HFD; D10080402, Research Diets, Inc., New Brunswick, NJ, USA) or a 10 kcal % fat diet (LFD; D10080401; Research Diets, Inc., New Brunswick, NJ, USA) for 12 weeks. Diet compositions are detailed in Table S1. The level of 45% fat by calories was chosen as it is commonly used to promote obesity in rodents without lowering carbohydrates to levels that would promote ketogenesis [3]. Body weights were recorded weekly. Food efficiency was calculated by dividing the body weight gained on a weekly basis by the amount of weekly food intake per animal, multiplied by the calories per gram of experimental diet (3.9 kcal/g LFD and 4.7 kcal/g HFD) to give the weekly caloric intake consumed (i.e., Food efficiency = Weekly BW gained/(Weekly food intake × Calories/g diet)]. Fat and fat-free mass measurements were obtained by Magnetic Resonance Imaging (MRI) (Echo Medical Systems, LLC., Houston, TX, USA) 3 days prior to starting the feeding intervention and 3 days prior to euthanasia. All animal experimental procedures were approved by the Rutgers University Animal Care and Use Committee.

### 2.2. Adipocyte Size and Number

Adipose tissues from 16 h fasted animals were dissected rapidly and fixed in 10% neutral buffered formalin (NBF, Avantik Biogroup, Pine Brook, NJ, USA). Fixed samples were embedded in paraffin, sliced in 5 μm thick sections, and stained with hematoxylin and eosin (H&E). For collagen detection, sections were stained with Masson’s trichrome reagent. Immunostaining was performed by Research Pathology Services (Rutgers University Biomedical Research Innovation Cores, Piscataway, NJ, USA). Images were acquired with an Olympus VS120-S5-W virtual slide microscope (Olympus Scientific Solutions Americas Corp., Waltham, MA, USA) and processed with OlyVIA Ver.2.9.1 viewer software. Morphometric analysis of white and brown adipocytes was performed with Fiji/ImageJ 2.1.0/1.53c software [20] and its plugin Adiposoft 1.16 [21] was additionally used for semi-automatic evaluation of white adipocytes, whereas brown adipocytes were evaluated manually. For each fat depot, morphological data were collected from at least 200–300 adipocytes per animal from non-overlapping random fields. Dead adipocytes surrounded by crown-like structures (CLSs) were not sized. Average adipocyte volume (FCV) in picoliters (pl) per cell was calculated from surface area using standard mathematical formula for a sphere, (πd^3^)/6, and the weighted average adipocyte weight and adipocyte number per depot was calculated as described previously. The depot is assumed to be 80% lipid with the density of triolein (0.915 g/mL) FCN (millions) per depot was calculated by dividing depot lipid weight by average adipocyte weight [(depot weight (g) × 0.8)/(mean adipocyte weight (mg lipid/cell) [13,22,23].

### 2.3. Preparation of Tissue and RNA Isolation

At the end of the feeding period, mice were food-deprived for indicated times, anesthetized with ketamine/xylazine/acepromazine (80:100:150 mg/kg, intraperitoneally, respectively), and their tissues were collected. Mice were euthanized under anesthesia. Inguinal and epididymal white and interscapular brown adipose tissue (iWAT, eWAT, and iBAT, respectively) depots from mice fasted for 4 h were treated with RNA*later*™ stabilization solution (Invitrogen™, Thermo Fisher Scientific, Waltham, MA, USA) upon excision before snap freezing in liquid nitrogen (N_2_) and storage at −80 °C.

Total mRNA was extracted from iWAT and iBAT depots using the PureLink™ RNA Mini Kit (Invitrogen™, Thermo Fisher Scientific, Waltham, MA, USA). All tissue samples were first homogenized using TRIzol™ reagent (Invitrogen™, Thermo Fisher Scientific, Waltham, MA, USA) for total RNA isolation. RNA abundance and quality were assessed using a NanoDrop 2000 Spectrophotometer (Thermo Fisher Scientific, Waltham, MA, USA). Two micrograms of total RNA were reverse-transcribed using the High-Capacity cDNA Reverse Transcription Kit with RNase Inhibitor (Applied Biosystems™, Thermo Fisher Scientific, Waltham, MA, USA).

### 2.4. RNA Sequencing Analysis

Total cellular mRNA was extracted as described above and RNA extracts derived from the iWAT of WT and *LFABP*^−/−^ mice were submitted for RNA-Sequencing (RNA-Seq) analysis to the Genomics Center of Rutgers New Jersey Medical School (Newark, NJ, USA). RNA quality was first checked for integrity using an Agilent 2200 TapeStation (Agilent Technologies, Inc., Santa Clara, CA, USA); samples with RNA integrity number (RIN) > 7.0 were used for subsequent processing. Total RNA was subjected to two rounds of poly(A) selection using oligo-d(T)25 magnetic beads (New England Biolabs, Ipswich, MA, USA). An Illumina-compatible RNA-seq library was prepared using a NEB next ultra RNA-seq library preparation kit. The cDNA libraries were purified using AmpureXP beads (Beckman Coulter Life Sciences, Indianapolis, IN, USA) and quantified on an Agilent TapeStation (Agilent Technologies, Inc., Santa Clara, CA, USA) and Qubit 4 Fluorometer (Thermo Fisher Scientific, Waltham, MA, USA). An equimolar amount of barcoded libraries was pooled and sequenced on the Illumina NovaSeq platform (Illumina, San Diego, CA, USA) using the 1 × 100 cycles configuration. CLC Genomics Workbench 20.0.4 version (http://www.clcbio.com/products/clc-genomics-workbench/ (accessed on 23 September 2021)); Qiagen (Redwood City, CA, USA) was used for RNA-seq analysis. De-multiplexed fastq files from RNA-Seq libraries were imported into the CLC software. Bases with low quality were trimmed and reads were mapped to the reference Mus musculus genome GRCm38. The reference genome sequence and annotation files were downloaded from ENSEMBLE, release.92 (Mus_musculus.GRCm38.92.fa, and Mus_musculus.GRCm38.92.gtf). The aligned reads were obtained using the RNA-Seq Analysis Tool of CLC Genomics Workbench using default settings (GEO Series number: GSE277001). Comparison of samples was performed using the gene set enrichment analysis (GSEA) software (v20.3.x), provided from the Broad Institute (https://www.gsea-msigdb.org/gsea/index.jsp, accessed on 14 May 2023), run on the GenePattern platform (https://www.genepattern.org/, accessed on 14 May 2023) and following the GSEA User Guide (http://www.gsea-msigdb.org/gsea/doc/GSEAUserGuideFrame.html, accessed on 14 May 2023) [24,25]. From the Metabolic Signatures Database (MSigDB, v7.5.1) (https://www.gsea-msigdb.org/gsea/msigdb/index.jsp, accessed on 14 May 2023) available, the KEGG, HALLMARK, and REACTOME gene set collections were used, which are coherently expressed signatures derived by aggregating many metabolic signature database (MSigDB) gene sets to represent well-defined biological states or processes [26,27]. For each gene set, a normalized enrichment score (NES) and a false discovery rate (FDR) q-value were generated. Five independent replicates for each group were used for analysis of differential expression. Differentially expressed pathways and genes with expression values > 20 Reads Per Kilobase per Million mapped reads (RPKM), FDRq value < 0.1 and fold change |FC| > 1.2 were used for downstream analysis.

Differentially expressed genes (DEGs) were identified from RNA-seq data using DESeq2 [28] with thresholds of |log2 fold change| > 1 and FDR < 0.1. To focus on functionally interpretable interactions, only protein-coding DEGs were retained. The list of protein-coding DEGs was submitted to the STRING database (version 12.0) [29] to predict potential protein–protein interactions. A minimum confidence score of 0.40 (medium confidence) was applied to filter spurious interactions. Proteins lacking interactions with other nodes were removed to enhance network interpretability.

### 2.5. Adipose Cholesterol Determination

Adipose samples were homogenized in 10 mM phosphate-buffered saline (PBS), as previously described [30]. Lipids were extracted using chloroform-methanol (2:1 *v*/*v*) by the method of Folch et al. [31]. Lipid extracts from known amounts of tissue and a 5-point concentration gradient using authentic standards were spotted on K5 Silica Gel 150 A TLC plates (Whatman #4852-820, Colonial Scientific, Richmond, VA, USA) and developed using a nonpolar solvent system (hexane-diethyl ether-acetic acid, 70:30:1 *v*/*v*). The plates were dried thoroughly and exposed to iodine vapors for 20 min to visualize and identify the lipid spots. Mass densitometry was analyzed using Fiji/ImageJ [20]. Data are expressed as mg cholesterol/g adipose tissue.

### 2.6. Immunohistochemical (IHC) Staining for Macrophage Infiltration

Sections were deparaffinized, dehydrated through a graded ethanol series, and subjected to heat-induced epitope retrieval with citrate buffer, pH 6.0 for 20 min at 98 °C using a pressure cooker. Primary antibody rabbit monoclonal anti-F4/80 (Cell Signaling Technology cat# 70076, Cell Signaling Technology, Danvers, MA, USA) was applied to sections at dilution of 1:750 for 1 h followed by an incubation in the secondary antibody Horse anti-Rabbit IgG Polymer (Vector MP6401, Vector Laboratories, Inc., Newark, CA, USA) for 30 min. DAB (3,3′Diaminobenzidine) chromogen substrate (Vector Labs SK-4105, Vector Laboratories, Inc., Newark, CA, USA) was added for 5 min for the development of a brown color followed by 1 min in hematoxylin (Vector H-3404, Vector Laboratories, Inc., Newark, CA, USA) for a background blue color.

### 2.7. Statistical Analysis

The data are presented as mean ± S.D. For body composition, food intake, and adipocyte quality-related measurements, comparisons were made between WT and LFABP null mice for each experimental diet, as well as within the same genotype between LFD and HFD. Differences between the groups were assessed using two-way ANOVA. Post hoc comparisons were performed using the Tukey test. For gene expression analysis, comparisons were made only between the HF-fed WT and LFABP null mice using two-tailed unpaired Student’s *t*-test. The data were analyzed using GraphPad Prism Version 9.5.1 for macOS (GraphPad Software, San Diego, CA, USA). The results were considered statistically significant when *p* < 0.05.

## 3. Results

### 3.1. Lfabp Deficiency Drives Hyperplastic Expansion of Inguinal WAT (iWAT) During Obesity Development

**Figure 1:**
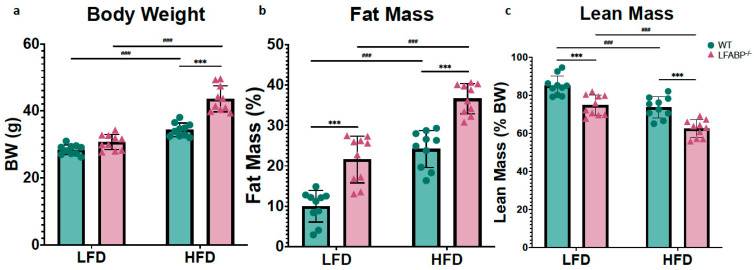

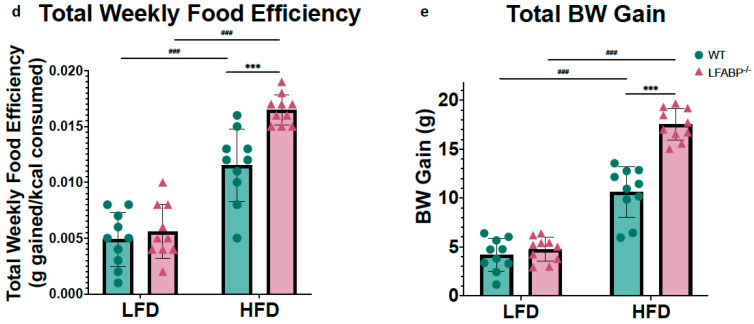
*Lfabp* deletion induces an obese phenotype. (**a**) Post-intervention body weight (g). (**b**) Lean mass (% of total BW). (**c**) Fat mass (% of total body weight, BW). (**d**) Mean weekly food efficiency per mouse (g). (**e**) Total body weight gain (g). *n* = 10. *LFABP*^−/−^ vs. WT: ***, *p* < 0.0001. HFD vs. LFD: ^###^, *p* < 0.0001. 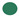 WT; 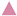 *LFABP*^−/−^. LFD, low-fat diet; HFD, high-fat diet; BW, body weight; WT, wild type; *LFABP*^−/−^, liver fatty acid-binding protein null.

As shown in Table 1, under LF feeding, *LFABP*^−/−^ and WT mice have similar body weight (BW); however, *LFABP*^−/−^ mice have 27% higher BW after 12 weeks of HFD (*p* < 0.001) (Figure 1a). Interestingly, body composition analysis indicated that even on LF feeding, the percent body fat of the *LFABP*^−/−^ mice was significantly higher compared to their WT counterparts (*p* < 0.001), a difference also found upon HF feeding (*p* < 0.001) (Figure 1b). While the percent lean mass was lower when compared with the WT mice (*p* < 0.001) (Figure 1c), the absolute lean mass remained unchanged (Table 1). Food efficiency was not different between the LF-fed WT and *LFABP*^−/−^ mice (*p* = 0.919) but was over 40% greater than WT in the HF-fed *LFABP*^−/−^ animals (*p* < 0.001) (Figure 1d). *Lfabp* ablation resulted in a 65% increase in total BW gain over the 12-week HFD intervention (*p* < 0.001) (Figure 1e).

**Figure 2:**
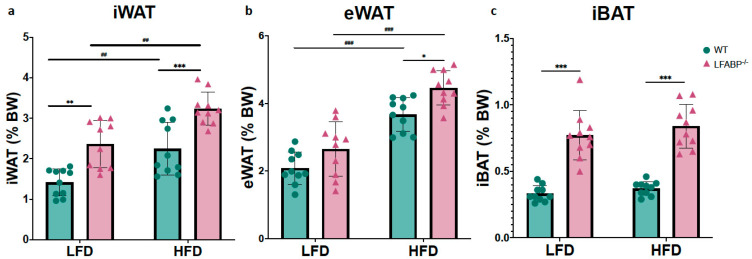
*Lfabp* deletion induces both white and brown adiposity upon DIO. Percentage contribution of (**a**) inguinal white adipose tissue (iWAT), (**b**) epididymal white adipose tissue (eWAT), and (**c**) interscapular brown adipose tissue (iBAT) to body weight (% BW) after LF and HF feeding. *n* = 10. *LFABP*^−/−^ vs. WT: *, *p* < 0.05, **, *p* < 0.001; ***, *p* < 0.0001. HFD vs. LFD: ^##^, *p* < 0.001; ^###^, *p* < 0.0001. 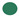 WT; 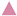 *LFABP*^−/−^. WT, wild type; *LFABP*^−/−^, liver fatty acid-binding protein null; LFD, low-fat diet; HFD, high-fat diet; BW, body weight; iWAT, inguinal white adipose tissue; eWAT, epididymal white adipose tissue; iBAT, interscapular brown adipose tissue.

**Figure 3:**
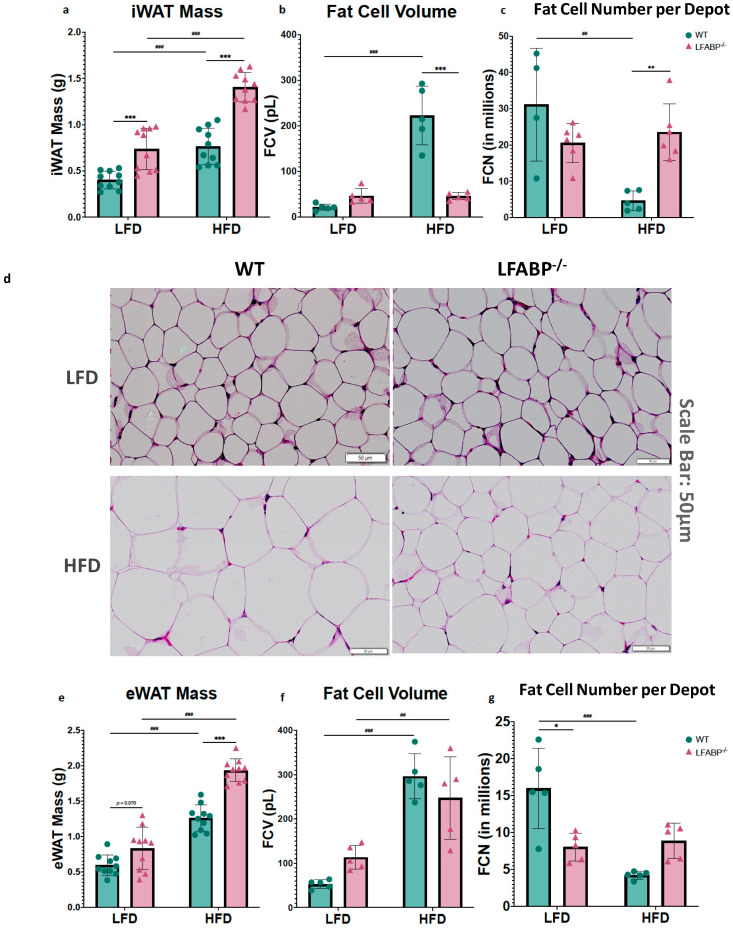

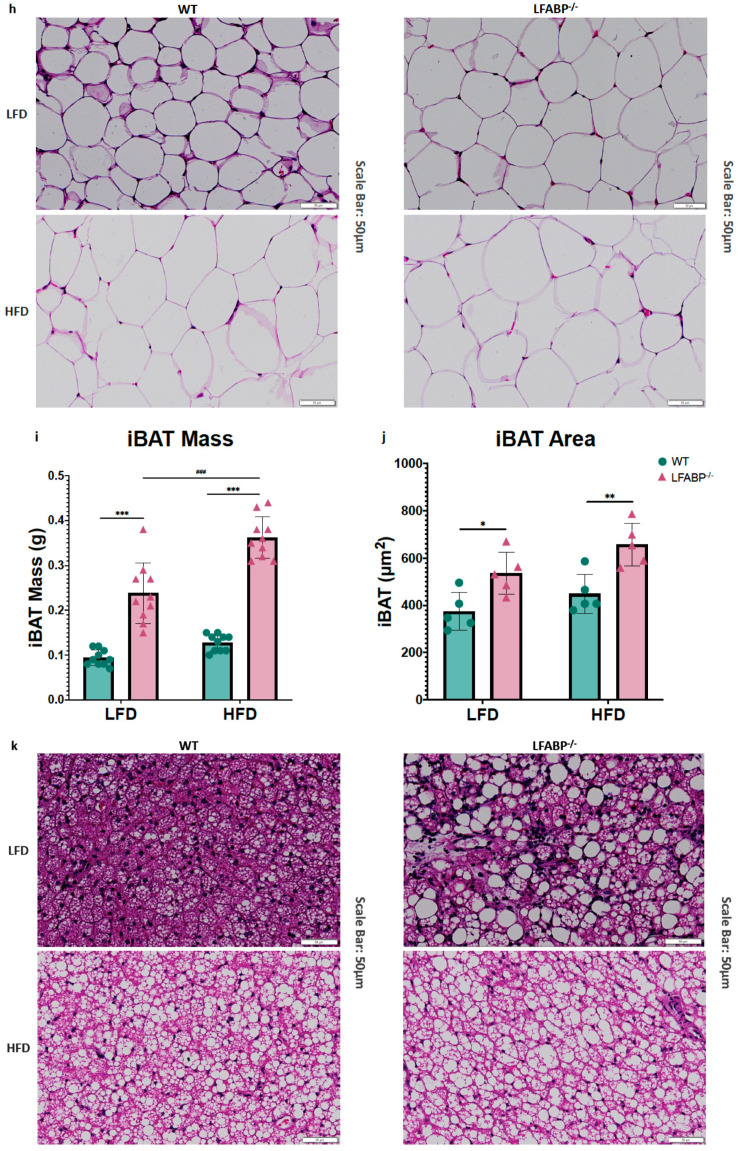
*Lfabp* deletion induces an altered adipocyte phenotype in subcutaneous, visceral, and brown adipose tissue depots. (**a**) Inguinal white adipose tissue (iWAT) mass (g) (*n* = 10). (**b**) Inguinal fat cell volume (FCV; pL) (*n* = 5). (**c**) Inguinal fat cell number (FCN) per depot (millions) (*n* = 5–6). (**d**) Representative H&E-stained pictures of iWAT depots. Scale bar, 50 μm. (**e**) Epididymal white adipose tissue (eWAT) mass (g) (*n* = 10). (**f**) Epididymal fat cell volume (FCV; pL) (*n* = 5). (**g**) Epididymal fat cell number (FCN) per depot (millions) (*n* = 5–6). (**h**) Representative H&E-stained pictures of eWAT depots. (**i**) Interscapular brown adipose tissue (iBAT) mass (g) (*n* = 10). (**j**) Brown adipocyte area (μm^2^) (*n* = 5). (**k**) Representative H&E-stained pictures of iBAT depots. Scale bar, 50 μm. *LFABP*^−/−^ vs. WT: *, *p* < 0.05; **, *p* < 0.001; ***, *p* < 0.0001. HFD vs. LFD: ^##^, *p* < 0.001; ^###^, *p* < 0.0001. 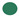 WT; 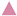 *LFABP*^−/−^. LFD, low-fat diet; HFD, high-fat diet; WT, wild type; *LFABP*^−/−^, liver fatty acid-binding protein null; iWAT, inguinal white adipose tissue; eWAT, epididymal white adipose tissue; FCV, fat cell volume; FCN, fat cell number; iBAT, interscapular brown adipose tissue.

In this study, iWAT served as a bona fide sWAT and eWAT as a vWAT depot, while iBAT served as a bona fide brown fat depot. On LF feeding, the inguinal white fat depot was 71% heavier in the null mice (iWAT: *p* = 0.001), whereas the epididymal depots were not different (eWAT: *p* = 0.160). Upon HF feeding, both iWAT and eWAT depots were significantly higher in the *LFABP* knockout mice (iWAT: *p* < 0.001; eWAT: *p* = 0.024) (Figure 2 and Table 1). Thus, *Lfabp* ablation resulted in higher absolute levels of iWAT mass, which was exacerbated under HF feeding, reaching almost double the mass of the WT mice (HFD: *p* < 0.001) (Figure 3a), in agreement with previous results [3]. The iBAT mass was >2-fold higher in the *LFABP* null mice relative to WT on both diets (*p* < 0.001) (Figure 2 and Table 1).

To determine the cellular basis of the markedly increased adiposity in *LFABP*^−/−^ mice, we measured cell diameters from each genotype for at least 200 randomly chosen inguinal, epididymal and interscapular brown adipocytes. Diameters for inguinal and epididymal fat cells were converted into cell volumes considering white adipocytes as spheres. LF-fed *LFABP*^−/−^ and WT mice were found to have comparable inguinal adipocyte size (fat cell volume, FCV; below 50 pL/cell) (*p* = 0.642). Upon HF feeding, the increase in iWAT mass was associated with a 4–5-fold increase in adipocyte size in the WT mice, as expected (Figure 3a,b) (*p* <0.001). Surprisingly and in marked contrast to WT, the HF-fed *LFABP*^−/−^ mice maintained an iWAT adipocyte size similar to those on the LFD (*p* > 0.999), and thus had a dramatic 4-fold smaller inguinal adipocyte size (*p* < 0.001) (Figure 3b) and 5-fold higher numbers of adipocytes in the iWAT depot (*p* = 0.001) (Figure 3c,d) when compared with their WT counterparts. By definition, therefore, the KO undergoes hyperplastic expansion, with an increased number of mature adipocytes per depot.

The *LFABP*^−/−^ and WT mice had comparable eWAT mass as a % of BW on LF feeding (*p* = 0.070). While eWAT mass increased significantly for both genotypes when they were fed an HFD, *Lfabp* ablation resulted in markedly higher eWAT mass with DIO development (*p* < 0.001) (Figure 3e). Interestingly, unlike what was found in the iWAT depot, the increase in eWAT mass observed on HF feeding was associated with an increase in adipocyte size in the *LFABP*^−/−^ mice, similar to the WT mice (*LFABP*^−/−^: *p* = 0.007; WT: *p* < 0.001) (Figure 3f). Notably, however, the increase in eWAT mass on HF feeding was accompanied by almost double the number of adipocytes in the *LFABP*^−/−^ mice, relative to WT (Figure 3g,h), although this did not reach statistical significance.

*Lfabp* ablation resulted in substantially larger iBAT mass and brown adipocyte area, relative to the WT, regardless of the feeding intervention (iBAT mass: *p* < 0.001 for both diets; brown adipocyte area: LFD, *p* = 0.038 and HFD, *p* = 0.007) (Figure 3i–k). Upon HFD feeding, *Lfabp* ablation resulted in greater iBAT mass relative to LF feeding (*p* < 0.001), whereas iBAT mass remained unchanged in the WT strain, independent of dietary fat intake (Figure 3i). It was noted that the brown adipocytes of the *LFABP* null mice appeared to be infiltrated by a higher lipid content, giving to the whole tissue a lighter appearance (Figure 3k).

### 3.2. Differential Pathway Enrichment in Subcutaneous Adipose Tissue upon Lfabp Ablation and HFD-Induced Obesity Development

To gain insight into the remarkable histological phenotypes noted in the iWAT, we performed RNA-seq followed by HALLMARK, KEGG, and REACTOME pathway enrichment analyses after 12 wks of HF feeding. The complete list of up- and downregulated pathways for all three MSigDBs; the volcano plot for REACTOME pathway enrichment analysis in the iWAT of HFD-fed WT and *LFABP*^−/−^ mice; and the STRING analysis-generated diagram showing known and predicted protein–protein interactions of DEGs can be found in Supplementary Materials (Tables S2 and S3 and Figures S1 and S2).

**Figure 4:**
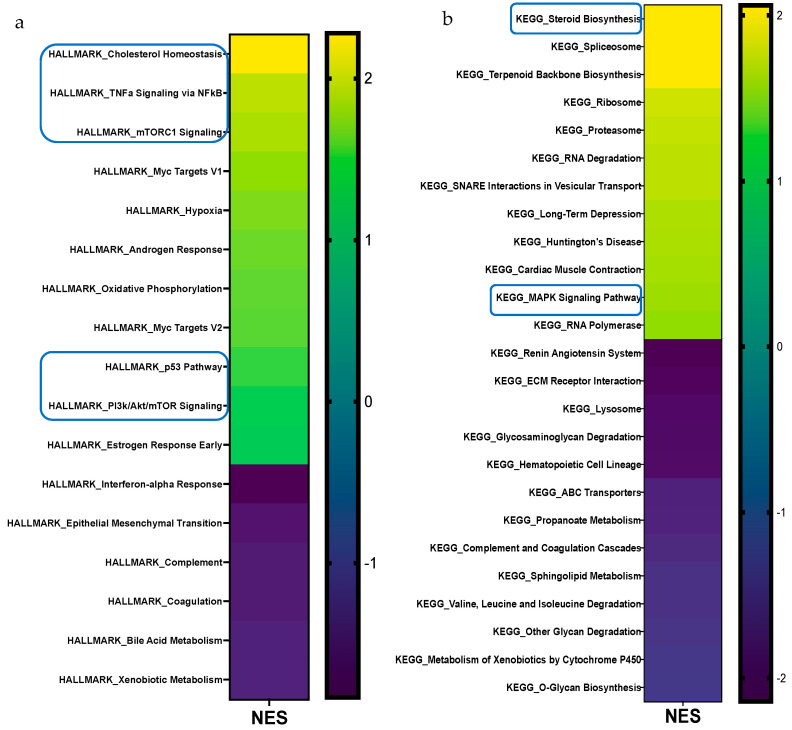

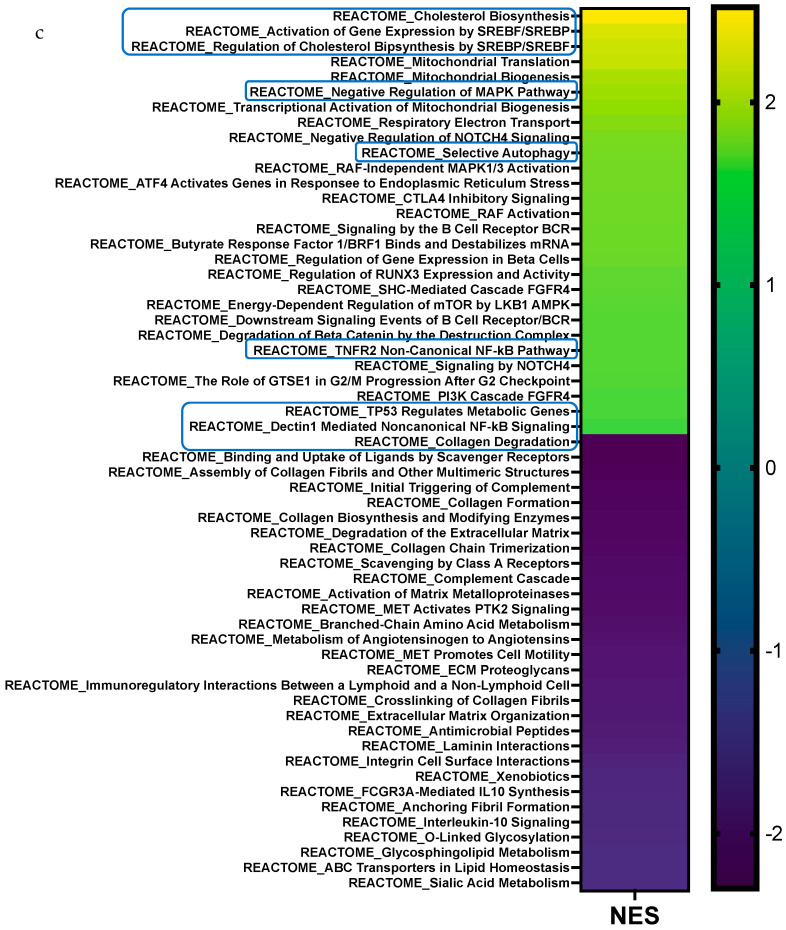
Gene set enrichment analysis (GSEA) of differentially expressed pathways in the iWAT of *LFABP* null mice upon HF feeding. The heatmaps present pathways that are significantly different for the MSigDBs (**a**) HALLMARK, (**b**) KEGG, (**c**) and REACTOME (*n* = 5/genotype). The direction and significance of each pathway were based on the gene set as a whole, while considering |NES| > 1.2 and FDRq value < 0.1. The boxes indicate pathways that are common between two or all three MSigDBs. MSigDB, molecular signature database; NES, normalized enrichment score; FDR, False Discovery Rate.

REACTOME pathway enrichment analysis in iWAT showed that among 1026 identified pathways (25,254 identified genes), 99 gene sets (137 transcripts) were differentially expressed between the *LFABP*^−/−^ and WT mice (FDRq < 0.1), with 61 upregulated (74 transcripts) and 38 downregulated (63 transcripts) in the HF-fed *LFABP*^−/−^ mice (|FC| > 1.2). We observed consistent upregulation of cholesterol metabolism-related pathways, multiple pathways involved in NF-*κ*B signaling, and mTORC1 and PI3K/Akt/mTOR signaling (Table 2 and Figure 4a–c). Downregulated gene sets in the HF-fed *LFABP*^−/−^ iWAT included ECM- and inflammation-related pathways (Table 2 and Figure 4a–c).

### 3.3. Lfabp Ablation Induces Distinct Transcriptional Responses in iWAT upon DIO

**Figure 5:**
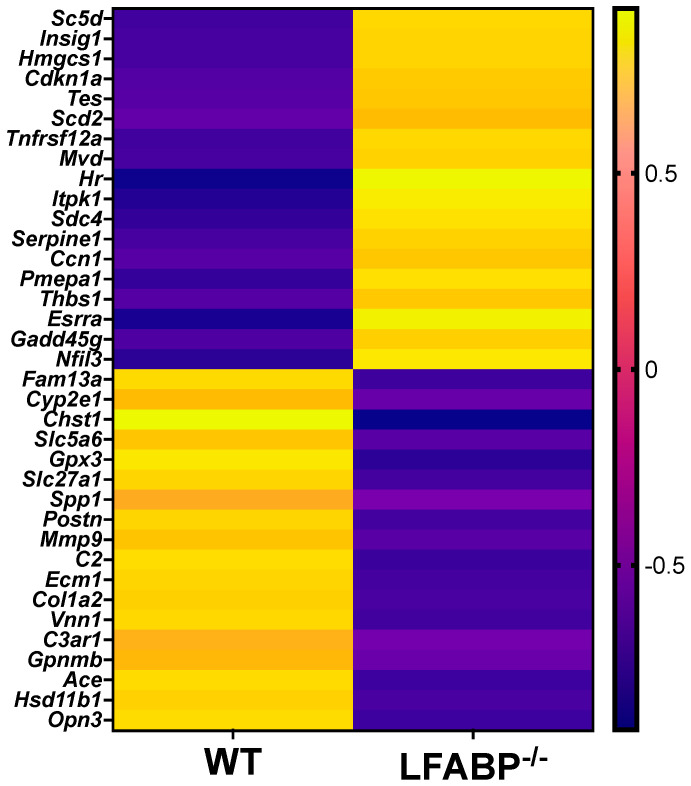
*Lfabp* ablation induces alterations in the transcriptomic profile of iWAT upon HF feeding. Heatmap of differentially expressed transcripts with RPKM values > 20, FDRq value < 0.1, and |FC| > 1.2 between *LFABP* null and WT mice (*n* = 5/genotype). FDR, false discovery rate; RPKM, Reads Per Kilobase per Million reads mapped.

We sought to evaluate the potential molecular basis for the marked increase in iWAT fat accumulation and unexpected higher cellularity upon obesity development and LFABP deficiency by interrogating the gene expression signature of the iWAT depot to identify changes in genes related to growth and obesity status. Among 25,254 identified genes, 137 transcripts were expressed differentially between *LFABP*^−/−^ and WT mice (FDRq < 0.1), with a total of 74 upregulated and 63 downregulated in the HF-fed *LFABP*^−/−^ mice (|FC| > 1.2). The top upregulated and downregulated differentially expressed genes are shown in Figure 5 and Table 2.

### 3.4. Lfabp Null Mice Show Altered Adipogenic Potential

**Figure 6:**
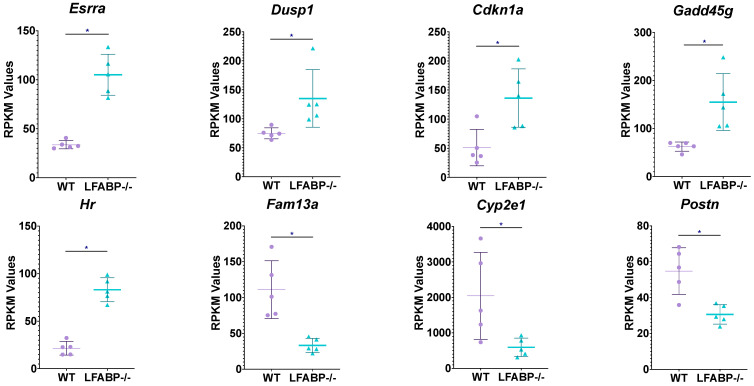
*Lfabp* deletion modifies the expression of genes reportedly related to adipogenesis. RNAseq reveals upregulated transcript levels for *Esrra*, *Insig*1, *Hr*, *Cdkn*1*a*, and *Gadd*45*g*, and downregulated transcript levels for *Cyp*2*e*1, *Fam*13*a*, and *Postn*, involved in cellular processes that may contribute to hyperplastic adipose tissue expansion in the iWAT of HF-fed *LFABP* null mice relative to the WT (*n* = 5 per genotype). FDRq < 0.1, |FC| > 1.2, RPKM > 20. *LFABP*^−/−^ vs. WT: *, *p* < 0.01. WT, wild-type; *LFABP*^−/−^, liver fatty acid-binding protein null; RPKM, Reads Per Kilobase of transcript per Million reads mapped; *Esrra*, Estrogen-Related Receptor Alpha; *Dusp1*, Dual-Specificity Phosphatase 1; *Cdkn1a*, Cyclin-Dependent Kinase Inhibitor 1A; *Gadd45g*, Growth Arrest and DNA-Damage-Inducible 45 Gamma; *Hr*, Hairless; *Fam*13*a*, Family with Sequence Similarity 13 Member A; *Cyp2e1*, Cytochrome P450 Family 2 Subfamily E Member 1; *Postn*, Periostin.

Given the striking hyperplastic phenotype observed in the iWAT of the *LFABP*^−/−^ mouse model, we inspected individual transcripts proposed to denote enhanced adipogenesis. Interestingly, we found an approximately 3.5-fold downregulation of cytochrome P450 family 2 subfamily E member 1 (*Cyp2e1*), characteristic of adipogenesis regulator (Areg) cells that inhibit adipogenesis through the retinoic acid signaling pathway [32], as well as those of family with sequence similarity 13 member A (*Fam*13*a*), implicated in adipocyte size and fat distribution [33], in the iWAT of HF-fed *LFABP*^−/−^ mice relative to WT. Additionally, Hairless (*Hr*) expression, which is significantly higher in the *LFABP*^−/−^ mice upon DIO, is required for white adipocyte development both in vitro and in vivo and is thought to be proadipogenic [34]. The orphan nuclear receptor estrogen-related receptor α (*ESRRA*) is also upregulated in *LFABP*^−/−^ mice, as is the transcript level of dual-specificity phosphatase 1 (*Dusp1*); these are known to be induced during adipocyte differentiation [35] and early proliferation of adipocyte progenitors [36], respectively; thus, their upregulation may indicate enhanced adipogenic capacity in the *LFABP*^−/−^ iWAT depot relative to WT. The transcript levels of genes involved in growth arrest, which is a crucial and necessary step during adipogenesis, are also upregulated in the HF-fed *LFABP* null mice, including *Cdkn*1*a* and *Gadd*45*g* (Figure 6 and Table S2).

### 3.5. Lfabp Deficiency May Enhance Cholesterol Biosynthesis and Subsequent mTORC1-Mediated iWAT Growth

**Figure 7:**
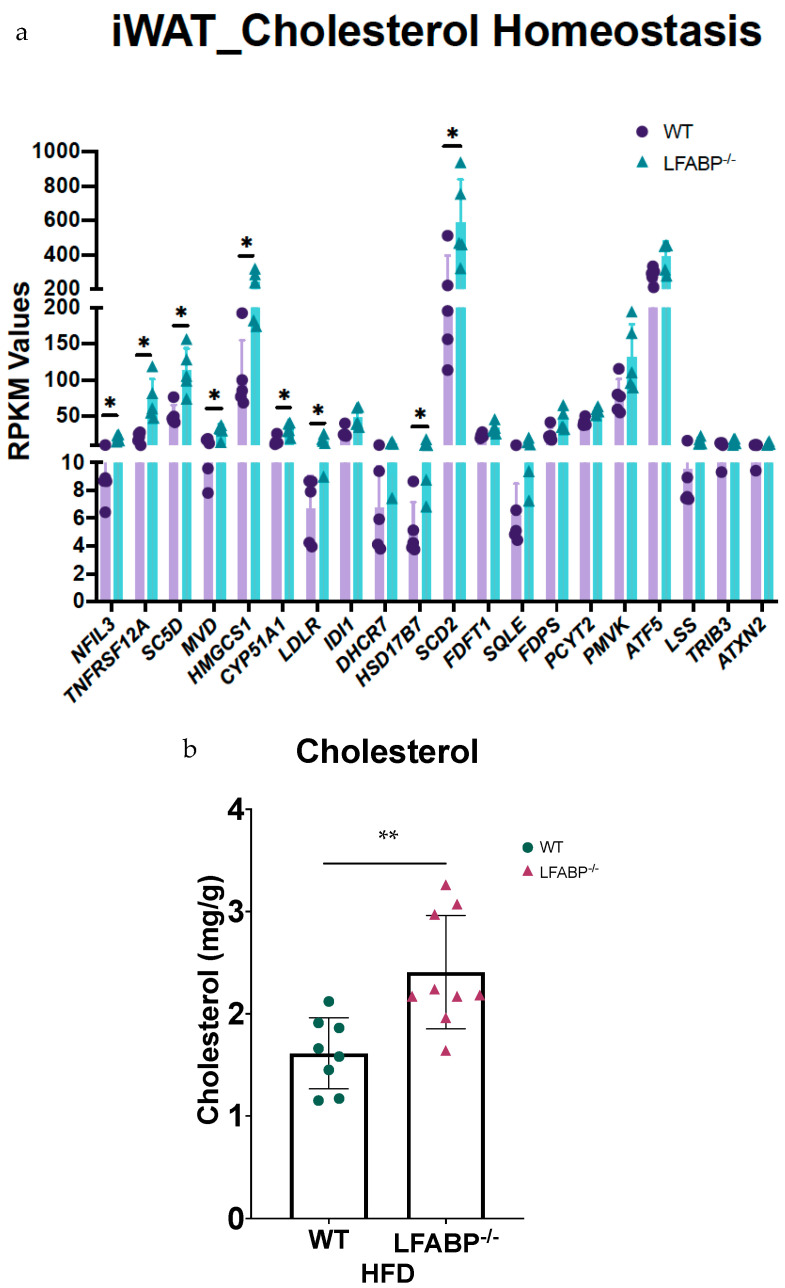
Upregulation of cholesterol metabolism genes and increased cholesterol content of iWAT upon *Lfabp* ablation and HFD challenge. (**a**) Gene expression; *n* = 5 per genotype. *LFABP*^−/−^ vs. WT: *, *p* < 0.01. (**b**) Free cholesterol content; WT, *n* = 8; *LFABP*^−/−^ *n* = 9. *LFABP*^−/−^ vs. WT: **, *p* < 0.001. 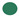 WT, wild type; 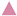 *LFABP*^−/−^, liver fatty acid-binding protein null; HFD, high-fat diet; iWAT, inguinal white adipose tissue.

All two MSigDBs used for RNA-seq pathway analyses reveal 2- to 3-fold increases in cholesterol biosynthesis and homeostasis pathways in the iWAT of HF-fed *LFABP*^−/−^ mice (Figure 4). Indeed, approximately 2- to 2.5-fold increases in the activation of cholesterol biosynthesis and SREBP gene expression pathways were found in the *LFABP*^−/−^ mouse model upon DIO (FDRq < 0.001 for both) (Figure 7a). The expression levels of 3-hydroxy-3-methylglutaryl-CoA synthase 1 (*Hmgcs*1) (FDRq = 0.036) were increased in the *LFABP*^−/−^ iWAT, suggesting upregulation of the early steps of cholesterol biosynthesis. The transcript levels of *Insig*1 were also upregulated in the HF-fed *LFABP*^−/−^ mice, possibly acting as a brake on cholesterol biosynthesis. Intriguingly, the PI3K/Akt/mTOR signaling pathway was enriched by 33% and that of the protein kinase mechanistic target of rapamycin complex 1 (mTORC1), which is involved in cell growth and cell survival [37] and has been shown to be activated by cholesterol [37], was enriched by 86% in the HF-fed *LFABP*^−/−^ mice relative to WT (Table 2). This is consistent with evidence demonstrating that increased INSIG1 is rapidly compensated by the activation of mTORC1 to restore SREBP1-mediated de novo lipogenesis gene expression [38]. Evidence of increased lipid synthesis more generally is seen in the 2.5-fold enrichment in the stearoyl-CoA-desaturase 2 (*Scd*2) transcripts in the HF-fed *LFABP*^−/−^ relative to WT mice (FDRq = 0.071) (Figure 7a). Importantly, we found that free cholesterol levels in the iWAT of the HF-fed *LFABP*^−/−^ mice were 50% higher compared to those of the WT mice (*p* = 0.003) (Table 1 and Figure 7b), corroborating the gene expression profiling results. STRING analysis of protein-coding genes shows the active nodes between DEGs involved with cholesterol biosynthesis pathways (Figure S2).

### 3.6. Lfabp Deletion Induces iWAT Immune and Inflammatory Responses and ECM and Angiogenesis Remodeling

The TNFα signaling pathway via NF-*κ*B, Dectin1-mediated noncanonical NF-*κ*B signaling, and the TNFR2 noncanonical NF-*κ*Β pathway, all involved in cellular immune and inflammatory pathways, are 1.9- (HALLMARK: FDRq <0.001), 1.7- (REACTOME: FDRq = 0.095), and 1.7-fold (REACTOME: FDRq = 0.092) enriched, respectively, in the HF-fed *LFABP*^−/−^ mice, relative to WT. In particular, the iWAT transcript levels of TNF receptor superfamily member 12A (*Tnfrsf*12*a*) are approximately 4-fold higher (FDRq < 0.001); TNFRSF12A serves as the specific receptor of the TNF-like weak inducer of apoptosis (TWEAK or TNFSF12A) [39,40,41,42] and is involved in noncanonical NF-*κ*B signaling.

Changes in multiple ECM-related pathways were found in the iWAT of HF-fed *LFABP*^−/−^ mice, including collagen formation and degradation, the biosynthesis of collagen and modifying enzymes, the assembly of collagen fibrils and other multimeric structures, the crosslinking of collagen fibrils, the degradation of the ECM, the activation of matrix metalloproteinases, ECM proteoglycans, ECM organization, and laminin interactions (Table 2, Table S2 and Figure 4b,c).

**Figure 8:**
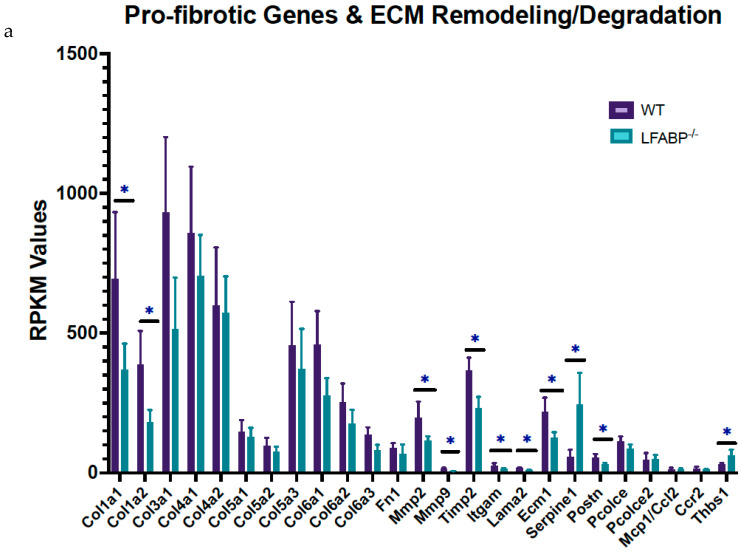

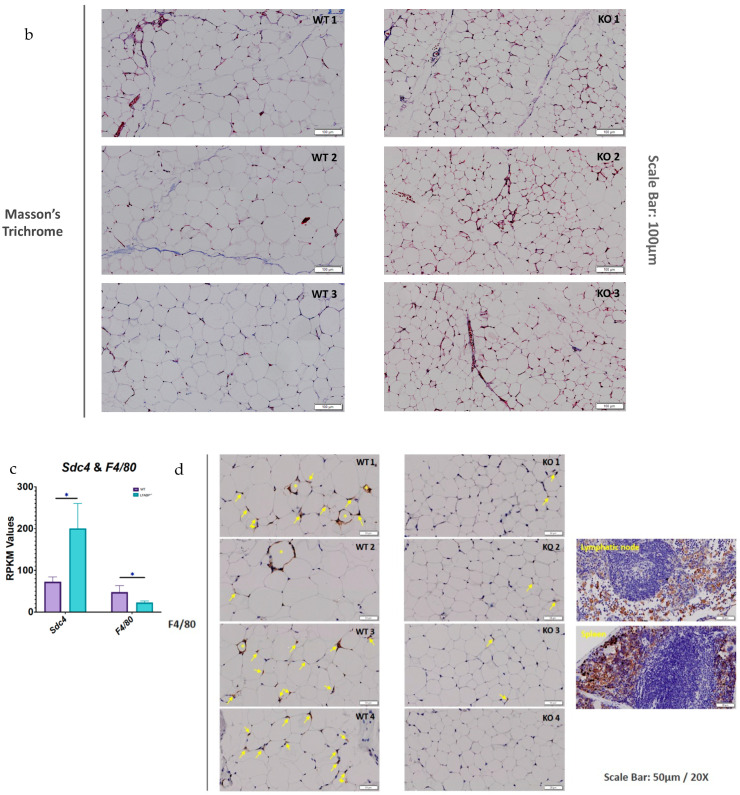
*Lfabp* deletion changes ECM remodeling dynamics, possibly causing adaptive fibrosis and protecting against inflammation in subcutaneous white adipocytes upon DIO. (**a**) RPKM values of pro-fibrotic and ECM remodeling/degrading components in the iWAT of *LFABP*^−/−^ mice relative to WT (*n* = 5 per genotype). (**b**) Representative Masson’s Trichrome staining of iWAT sections in HF-fed WT and *LFABP*^−/−^ mice. Magnification: 20×. Scale bar, 100 μm (*n* = 3 per genotype, from different animals). (**c**) RPKM values of *Sdc*4 and *F*4/80 in HF-fed *LFABP*^−/−^ mice relative to WT (*n* = 5 per genotype). (**d**) Representative images of IHC staining for F4/80 in the HF-fed *LFABP*^−/−^ iWAT compared to the WT. The stars show positive cells organized in CLS around an adipocyte; the arrows show positive cells without specific organization. Positive control for mesenteric lymphatic node and spleen specimens. Magnification: 20×. Scale bar, 50 μm (*n* = 4 per genotype, from different animals). *LFABP*^−/−^ vs. WT: *, *p* < 0.01. WT, wild type; *LFABP*^−/−^, liver fatty acid-binding protein null; KO, knockout; *Sdc*4, Syndecan 4; *F*4/80/*Adgre*1, Adhesion G Protein-Coupled Receptor E1; CLS, Crown-like structure; RPKM, Reads Per Kilobase of transcript per Million reads mapped.

The transcriptomic profile of iWAT reveals that, among the ECM components, levels of several collagens (*Col*1*a*1, *Col*1*a*2, *Col*3*a*1), along with *Postn* (periostin), *Lama*2 (laminin α-2 chain), *Spp*1 (osteopontin), and *Ecm*1 (extracellular matrix protein 1), are significantly downregulated, whereas *Thbs*1 (thrombospondin-1) is significantly upregulated in HF-fed *LFABP*^−/−^ mice relative to WT (Figure 8a). The expression level of *Adamts*12, an ECM-constructive enzyme, was downregulated in the *LFABP*^−/−^ mouse model upon DIO, possibly indicating decreased collagen levels due to impaired proteolytic cleavage. The expression levels of the degrading enzymes MMP2 (matrix metalloproteinase 2) and MMP9 were also downregulated, as was the level of their inhibitor TIMP2 (tissue metalloproteinase inhibitor 2), presumably explaining the similar transcript levels of the collagen types COL-4 and 5 between the *LFABP*^−/−^ and WT mice (Figure 8a). By contrast, expression levels of the serine protease inhibitor clade E member 1 (*Serpine*1), also known as plasminogen activator inhibitor-1 (PAI-1), are significantly elevated in the *LFABP*^−/−^ iWAT, possibly indicating inhibition of the ECM fibrinolytic system and, thus, ECM degradation (Figure 8a).

SERPINE1 has been shown to induce cell proliferation and migration, as well as pro-angiogenic activity [43,44,45,46]. Moreover, Masson’s trichrome staining for fibrosis shows no appreciable differences between the HF-fed *LFABP* null and WT mice. It is of interest, however, that small adipocytes are found close to more fibrotic areas in the KO iWAT sections, which is consistent with the concepts of ‘adaptive fibrosis’ and ‘homeostatic adipogenesis’ [47,48,49] (Figure 8b).

The transcript levels of *Sdc*4 (syndecan 4), involved in ECM interactions and vasculogenesis, are increased, whereas those of *F*4/80 (or *Adgre*1), characteristic of macrophage infiltration, are decreased in the HF-fed *LFABP*^−/−^ iWAT relative to WT (Figure 8c). To directly assess macrophage infiltration, iWAT sections were stained for F4/80; in keeping with the gene expression data, markedly decreased infiltration of macrophages in the subcutaneous adipose tissue of the MHO *LFABP*-ablated mice relative to the WT was observed, including large areas with no apparent macrophage staining at all. Those positive cells found in the iWAT of *LFABP*^−/−^ mice are mostly single, mildly stained isolated macrophages. In contrast, in the iWAT of WT mice, there were more areas showing the presence of macrophages, and the greater intensity of the staining indicates the aggregation of more cells compared to the iWAT of the KOs. Notably, whereas in the iWAT of HF-fed WT mice, we detected areas with positive cells organized in CLSs around adipocytes; CLSs were not found in the iWAT of the *LFABP*^−/−^ mice (Figure 8d), in keeping with the MHO phenotype of these mice. DEGs involved in ECM remodeling and noncanonical NF-κB signaling are visualized in the STRING analysis diagram revealing potential protein–protein interactions in the HF-fed *LFABP*^−/−^ iWAT (Figure S2).

Collectively, these results reveal that while the WT responds as expected to an HFD by developing inflamed adipose tissue with increased pericellular fibrosis, the *LFABP* KO manages to prevent this, showing neither inflammation nor macrophage infiltration in iWAT upon DIO.

## 4. Discussion

### 4.1. Evidence of Hyperplastic WAT Expansion upon Lfabp Deletion and HF Feeding

Subcutaneous adipose tissue is the largest and at the same time the least metabolically harmful AT depot in the body [15]. With the development of obesity, WAT undergoes a process of tissue remodeling in which adipocytes increase in both number and size [16,23,50,51]. Evidence suggests that the recruitment of APCs is a feature of healthy AT expansion to meet the need of storing excess energy. This process necessitates remodeling of the fibrous ECM [11,22], which may become a limiting factor for adipocyte size in the context of obesity [11].

LF-fed *LFABP*^−/−^ mice have higher fat mass compared to WT mice, with the size of white adipocytes similar under LF feeding. Remarkably, though, whereas the WT mice respond as expected when challenged with HFD, the *LFABP*^−/−^ iWAT depot becomes populated by approximately 5-fold-higher numbers of inguinal adipocytes, whose size is only 25% that of their WT counterparts. This marked increase in fat cells per depot shows an unusual hyperplastic expansion of subcutaneous fat in the HF-fed *LFABP*^−/−^ mice. Smaller adipocytes are thought to be more insulin-sensitive [9,16,50,52]; thus, the effect of *Lfabp* ablation on the quality of sWAT may contribute to the previously reported MHO phenotype of the *LFABP*^−/−^ mouse, which is characterized by normal plasma glucose and insulin levels despite marked obesity [3].

### 4.2. Lfabp Deletion Induces Alterations in the Transcriptomic Profile of Subcutaneous Fat Suggestive of an Enhanced Adipogenic Program

The significantly upregulated expression of several pathways, particularly those of cholesterol metabolism and biosynthesis, mTORC1, and PI3K/Akt/mTOR signaling, are indicative of enhanced tissue growth in HF-fed *LFABP*^−/−^ mice relative to WT. Moreover, the modulation of specific transcripts in the iWAT of HF-fed *LFABP* null mice relative to WT point to healthy AT expansion. For example, the decreased expression of *Cyp*2*e*1 is of particular interest, as its expression is enriched in CD142^+^ cells, a subpopulation of adipose stem and progenitor cells (ASPCs) that has been attributed non- and anti-adipogenic properties both in vivo and in vitro [32,53]. *Cyp*2*e*1-deficient mice, moreover, were shown to be protected against DIO and insulin resistance [54]. Thus, the robust downregulation of *Cyp*2*e*1 in the iWAT of *LFABP*^−/−^ mice may be indicative of enhanced adipogenic capacity and improved glycemic control, as found in these mice [4].

Lower levels of *Fam*13*a* were also found in the hyperplastic iWAT of HF-fed *LFABP*^−/−^ mice. A murine model of *Fam*13*a* deficiency exhibited higher numbers of small adipocytes in iWAT, enhanced adipogenic potential, and preserved glucose uptake and insulin responsiveness [33], findings that are very similar to those observed herein in HF-fed *LFABP*^−/−^ mice, suggesting that *Fam*13*a* downregulation may contribute to the hyperplastic iWAT phenotype in these mice.

The iWAT of HF-fed *LFABP* null mice was also found to have enriched *Esrra* transcripts relative to the WT. ESRRA deficiency in mice is characterized by reduced adiposity, possibly due to a decrease in APCs differentiation and adipogenesis in WAT [35,55,56]. Thus, the enriched *Esrra* transcript levels herein further support the observed increased cellularity and enhanced adipogenic potential in the HF-fed *LFABP*^−/−^ iWAT. Interestingly, ESRRA has been additionally shown to regulate the transcription of genes participating in autophagy [57], which is progressively induced during adipocyte differentiation, such that a loss of autophagy may compromise white adipogenesis [58,59,60,61]. Cholesterol has been shown to serve as an endogenous ligand for ESRRA [57,62,63,64,65]; thus, the significant increases found in cholesterol biosynthesis pathways and iWAT cholesterol levels in HF-fed *LFABP*^−/−^ mice may be related to increased *Esrra*-mediated adipogenesis.

The transcript levels of *Scd*2 were found in the *LFABP*^−/−^ mouse model. SCDs are well known to be induced during the differentiation of 3T3-L1 preadipocytes into adipocytes and in AT with obesogenic diets [66,67]. The monounsaturated fatty acids (MUFAs), products of SCDs, are critical components of cellular membrane phospholipids (PLs), cholesterol esters, and TAG stores, contributing to appropriate adipocyte membrane structure and function as well as sufficient capacity for excess energy storage, both of which would be necessary for cell proliferation and growth in newly emerging APCs in the obese *LFABP*^−/−^ mouse model. Overall, although the mechanisms driving the hyperplasia remain to be definitively determined, the transcriptomic changes found in iWAT largely support the observed hyperplastic growth of *LFABP*^−/−^ adipose tissue during HFD feeding.

### 4.3. Enrichment in Cholesterol-Related Pathways and Increased Cell Cholesterol Content May Contribute to Small, Insulin-Sensitive Adipocytes in the iWAT of HF-Fed LFABP^−/−^ Mice

The transcriptomic data showing a significant upregulation of cholesterol biosynthesis in the HF-fed *LFABP*^−/−^ mice, relative to the WT, are consistent with the observed higher tissue cholesterol levels. Interestingly, both the tissue levels and subcellular distribution of cholesterol are proposed to be involved in adipocyte cell size regulation, where the depletion of cholesterol from the plasma membrane has been reported to reproduce insulin resistance-related defects found in enlarged fat cells, possibly via the internalization of surface caveolins [48,68,69]. Increased sterols combined with the markedly smaller inguinal adipocyte size may suggest increased cholesterol in the plasma membrane relative to the lipid droplets [48], consistent with the maintained insulin sensitivity described previously in the MHO *LFABP*^−/−^ mouse model [3,4].

The increased levels of *Insig*1 expression in the iWAT of HF-fed *LFABP*^−/−^ mice are consistent with the elevated adipose tissue levels reported at the onset of DIO [70]. Interestingly, it has been shown that the expression of *Insig*1 progressively increases during the maturation of adipocyte progenitors and impedes lipogenesis in mature adipocytes, thereby determining adipocyte size and storage capacity [71]. It has also been reported that the INSIG1-mediated blockade of adipose tissue lipogenesis is immediately compensated by the activation of mTORC1 to restore SREBP1-dependent de novo lipogenesis gene expression [71]. This, too, is in accordance with the observed upregulation of the PI3K/Akt/mTOR and mTORC1 signaling pathways, as well as increases in the activation of gene expression by SREBP and cholesterol biosynthesis in the *LFABP*^−/−^ mouse model upon DIO. Cholesterol in turn can mediate the activation of the mTORC1 signaling pathway and its downstream anabolic effects, including cell growth, proliferation, metabolism, survival, and angiogenesis [37,72,73], as observed herein.

### 4.4. Lfabp Ablation Alters Immune and Inflammatory Responses in Inguinal Adipocytes upon DIO

Noncanonical NF-*κ*B signaling can be stimulated after lipopolysaccharide (LPS) binding to Dectin1 and TNFR2 and can be initiated by TNFR2 and TNFRSFs (TNFR superfamily members) such as TNFRSF12a, which is significantly enriched in the iWAT of HF-fed *LFABP*^−/−^ mice. Overexpression of TNFRSF12A has been shown to activate the noncanonical NF-*κ*B pathway independent of binding to its ligand TWEAK [74], and indeed, *Tweak* transcript levels are similar between the *LFABP*^−/−^ and WT mice. Bacterial LPS-induced inflammation may induce TNFRSF12A expression via TNFα signaling [75]; thus, the observed *Tnfrsf*12 overexpression may be induced by an extracellular stimulus, e.g., LPS derived from obesity-related gut bacterial species [76]. We have shown that LPS biosynthetic pathways are significantly upregulated in *LFABP*^−/−^ intestinal mucosa [77]. In addition to TNFR2, Dectin1, which has been shown to be involved in adaptive immunity and tolerance [78], is also significantly upregulated in the *LFABP* null mice.

It has been suggested that the inflammatory responses classically attributed to TNFα, including LPS-induced cytotoxicity, are mediated by TNFR1, whereas TNFR2 has been implicated in the suppression of TNFα-induced inflammatory responses and NF-*κ*B-dependent gene expression [79]. It is therefore possible that, despite their adiposity, *LFABP*^−/−^ mice may be, at least partially, protected against adverse inflammatory responses through the noncanonical activation of NF-*κ*B signaling through TNFR2 and Dectin1.

### 4.5. Lfabp Deletion May Alter Inguinal Fat ECM Remodeling, Fibrosis, Inflammation, and Angiogenesis upon DIO

A biphasic development of the ECM has been suggested to occur during adipogenesis, in which the fibrillar collagen types 1 and 3 are decreased early on and return to their initial levels at the later stages of differentiation [80,81,82]. In this study, HF-fed *LFABP*^−/−^ mice display downregulated expression of *Col*1*a*1, *Col*1*a*2, and *Col*3*a*1 in iWAT, possibly indicative of newly differentiated adipocytes that have not fully acquired the mature adipocyte phenotype. Intriguingly, *Postn* deficiency has been reported to attenuate LPS-/HFD-induced AT fibrosis and to improve IR; thus, the lower *Postn* levels found herein may contribute to healthy hyperplastic AT expansion and maintain insulin sensitivity during obesity [83,84], as we observed in the *LFABP*^−/−^ mouse model.

*Spp*1 encodes for osteopontin, an ECM protein important in cell adhesion, migration, and ECM degradation [85] and one that has been demonstrated to inhibit adipogenic and promote osteogenic differentiation in mesenchymal stem cells (MSCs) [86]. Here, we found transcriptional downregulation of *Spp*1 in the iWAT of the HF-fed *LFABP*^−/−^ mice. A deficiency of osteopontin results in increased ratios of both subcutaneous and visceral AT to body weight [86], as was found in the *LFABP*^−/−^ mice.

We also found increased *Sdc*4 transcript levels in the *LFABP*^−/−^ iWAT, compared to the WT, further corroborating that ECM remodeling may influence adipose tissue remodeling during obesity development. During DIO, *Sdc*4 deletion contributes to dyslipidemia, hyperglycemia and insulin resistance, as well as increased adipocyte size and macrophage infiltration [68]. Our findings in HF-fed *LFABP*^−/−^ mice, which overexpress *Sdc*4, are consistent with these results, since we find a marked decrease in the size of inguinal adipocytes; decreased expression and staining of F4/80, indicative of lower macrophage infiltration; and normal glycemic and lipidemic profiles despite massive adiposity, as previously reported [3]. This too confers additional support for low inflammation and maintained insulin sensitivity in the HF-fed *LFABP*^−/−^ mouse model.

Elevation of *Serpine*1 in the *LFABP*^−/−^ iWAT may indicate an inhibition of the ECM fibrinolytic system and, thus, ECM degradation. While the role of SERPINE1 in adipogenesis and angiogenesis is controversial [69,87,88,89,90], it has been shown that the overexpression of SERPINE1 in AT results in larger fat pads with higher adipocyte density [87], consistent with the phenotype found in the *LFABP*^−/−^ iWAT. Additionally, the inhibitory effect of *Serpine*1 on ECM degradation could, potentially, serve as a brake on adipocyte size. Indeed, excessive ECM deposition in AT during obesity development has been suggested to contribute to a state of adaptive fibrosis, in which a more rigid ECM may prevent excessive enlargement of adipocytes and preserve adipocyte function [47,49]. In this regard, APCs have been demonstrated to accumulate in fibrotic areas within the WAT of obese individuals [91]. HF-fed *LFABP*^−/−^ iWAT stained for Masson’s trichrome show that the numerous small adipocytes described here appear to be aggregated in regions with more intense staining. Scarce macrophage infiltration and negligible or absent CLS formation in the subcutaneous fat of the *LFABP*^−/−^ mice indicates a lower inflammatory state and maintained metabolic function within the depot, supporting the notion of an ‘adaptive fibrosis’ and a metabolically healthy obese state.

Overall, given the massive iWAT adiposity of the *LFABP*^−/−^ mouse model, we speculate that their inguinal adipocytes reach a critical/maximal size, after which hyperplastic growth is triggered earlier during the HF-feeding period, relative to WT. This would lead to APC recruitment, which, in turn, would regulate ECM remodeling to drive hyperplastic iWAT expansion. It has been reported that hypertrophy precedes hyperplasia/adipogenesis, and that adipocyte hypertrophy and turnover increase depending on the rate of change of overall fat mass [9,92]. Therefore, the fact that *LFABP*^−/−^ mice challenged with HF feeding become more obese compared with their WT counterparts, in combination with decreased ECM degradation that would render the ECM in iWAT more rigid, may offer a reasonable explanation for the presence of smaller adipocytes. Notably, the phenotype is different on LF feeding, where *Lfabp* ablation appears to predispose for higher iWAT mass relative to the WT, despite similar BW, and where adipocyte size is comparable to WT.

While the SAT hyperplastic phenotype is clear, as evidenced by the markedly greater number of fat cells per depot, it is worth noting that only male mice were studied in depth thus far. Additionally, subcutaneous WAT was thoroughly examined, whereas visceral WAT and interscapular BAT were not extensively studied apart from cellularity and histological features. Given the dramatic changes observed in BAT in particular, future studies will characterize physiological and transcriptional changes in BAT, which may contribute to the MHO phenotype of the obese *LFABP* null mice, including genes that regulate mitochondrial fission, which have been linked to increased cell proliferation, adipocyte differentiation, and ECM remodeling [93,94]. It will also be informative to examine the time course of adipogenesis in vivo and to further assess transcriptomic changes using single-cell RNA-seq to dissect mechanistic pathways at the adipocyte level that contribute to DIO-dependent hyperplastic expansion in the *LFABP*^−/−^ mouse.

## 5. Conclusions

**Figure 9:**
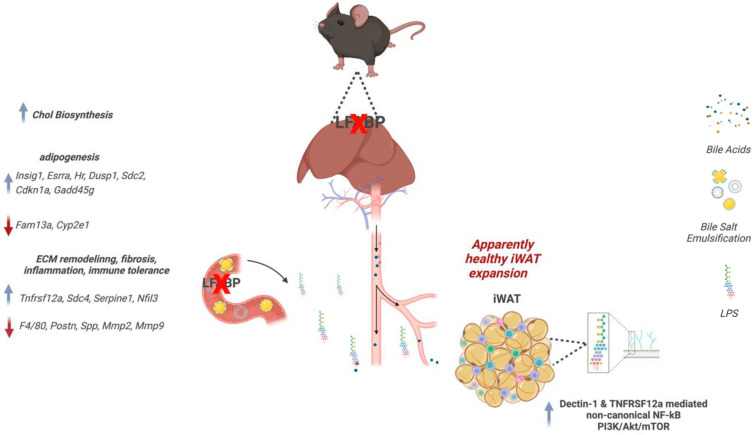
*Lfabp* deletion and HF feeding lead to the hyperplastic expansion of iWAT. Whole-body *Lfabp* ablation results in a heavier iWAT depot with more numerous and smaller adipocytes, i.e., hyperplastic iWAT expansion. Transcriptomic modifications of genes reportedly involved in enhanced adipogenesis, as well as ECM remodeling, inflammation, and immune tolerance, are suggested to participate in the observed adipocyte phenotype. Additionally, the upregulation of pathways involved in cholesterol biosynthesis, noncanonical NF-*κ*B signaling upon potential LPS-mediated Dectin-1 and TNFRSF12a stimulation, and PI3K/AKT/mTOR signaling is also proposed to contribute to hyperplastic iWAT growth upon *Lfabp* deletion and diet-induced obesity. Chol, cholesterol; ECM, extracellular matrix; iWAT, inguinal white adipose tissue; LFABP, liver fatty acid-binding protein; LPS, lipopolysaccharide; TNFRSF12a, Tumor Necrosis Factor Receptor Superfamily Member 12A.

In summary, we show that while WT animals respond as expected to DIO by developing hypertrophied inguinal adipocytes and inflamed iWAT with increased pericellular fibrosis, the *LFABP* null mouse model, by contrast, expands by increasing adipocyte number, not size, and is protected from inflammation and macrophage infiltration (Figure 9). Since LFABP is not expressed in AT, these results suggest that its deficiency promotes interorgan signaling that affects adipocyte cellularity, limiting hypertrophy and driving hyperplasia in the expansion of potentially metabolically beneficial subcutaneous iWAT. The previously described MHO phenotype of *LFABP* null mice, characterized by normal glucose and insulin levels as well as decreased hepatic steatosis [3] and resistance to obesity-induced decrease in exercise capacity [4], is further corroborated in the present study, where we show a highly unusual hyperplastic mode of subcutaneous fat expansion. The mechanisms by which LFABP, a lipid transport protein expressed within the liver and the proximal intestine, impacts AT and, as we showed previously, muscle tissue [4], remains an intriguing question. We found increased gut microbial diversity [76] and intestinal mucosal anandamide (AEA) levels [3] upon *Lfabp* ablation. Moreover, preliminary data show alterations in levels of other endocannabinoid-like lipids, as well as in plasma BA levels and BA species, and a large shift in the ratio of primary to secondary bile acids [77]. Since LFABP binds both bile acids and endocannabinoids, we speculate that changes noted in bile acid–gut microbiota metabolism and endocannabinoid system-related signaling may be involved in mediating the effects of *Lfabp* ablation on peripheral tissues and systemic metabolism.

## Figures and Tables

**Table 1:** Phenotypic comparison of wild-type and *LFABP* knockout mice on LFD and HFD.

Parameter	LFD		*p*-Value *LFABP*^−/−^ vs. WT	HFD		*p*-Value *LFABP*^−/−^ vs. WT	*p*-Value HFD vs. LFD	
	WT	*LFABP* ^−/−^		WT	*LFABP* ^−/−^		WT	*LFABP* ^−/−^
Week 12 BW, g (*n* = 10)	28.4 ± 1.5	30.7 ± 2.3	*p* = 0.212	34.4 ± 2.0	43.6 ± 3.9	*p* < 0.001	*p* < 0.001	*p* < 0.001
Weight gain over 12 wk, g (*n* = 10)	4.2 ± 1.7	4.8 ± 1.2	*p* = 0.888	10.6 ± 2.6	17.5 ± 1.6	*p* < 0.001	*p* < 0.001	*p* < 0.001
Fat mass, g (*n* = 10)	2.7 ± 1.1	6.7 ± 2.2	*p* < 0.001	8.3 ± 1.7	15.4 ± 2.3	*p* < 0.001	*p* < 0.001	*p* < 0.001
Fat mass, %BW (*n* = 10)	10 ± 3.9	21.6 ± 5.8	*p* < 0.001	24.2 ± 4.6	36.6 ± 3.7	*p* < 0.001	*p* < 0.001	*p* < 0.001
Adiposity Index, % (*n* = 10)	4.8 ± 0.9	8.2 ± 1.1	*p* < 0.001	8.2 ± 1.1	10.8 ± 0.8	*p* < 0.001	*p* < 0.001	*p* < 0.001
Lean mass, g (*n* = 10)	23.4 ± 2.1	22.9 ± 0.9	*p* = 0.899	25.3 ± 2.1	25.8 ± 1.8	*p* = 0.929	*p* = 0.112	*p* = 0.005
Lean mass, %BW (*n* = 10)	85.1 ± 5.2	74.8 ± 5.3	*p* < 0.001	73.7 ± 5.5	62.5 ± 4.6	*p* < 0.001	*p* < 0.001	*p* < 0.001
iWAT, g (*n* = 10)	0.4 ± 0.1	0.7 ± 0.2	*p* < 0.001	0.8 ± 0.2	1.4 ± 0.2	*p* < 0.001	*p* < 0.001	*p* < 0.001
iWAT, %BW (*n* = 10)	1.4 ± 0.3	2.4 ± 0.6	*p* = 0.001	2.3 ± 0.7	3.2 ± 0.4	*p* < 0.001	*p* = 0.005	*p* = 0.003
iWAT FCV, pL (*n* = 5)	21.4 ± 6.8	46.6 ± 16.3	*p* = 0.642	222 ± 64.1	45.6 ± 7.8	*p* < 0.001	*p* < 0.001	*p* >0.999
iWAT FCN per depot, millions (*n* = 5–6)	31.2 ± 15.6	20.6 ± 5.4	*p* = 0.244	4.7 ± 2.7	23.6 ± 7.8	*p* = 0.001	*p* = 0.009	*p* = 0.925
eWAT, g (*n* = 10)	0.6 ± 0.2	0.8 ± 0.3	*p* = 0.070	1.3 ± 0.2	1.9 ± 0.2	*p* < 0.001	*p* < 0.001	*p* < 0.001
eWAT, %BW (*n* = 10)	2.1 ± 0.5	2.7 ± 0.8	*p* = 0.160	3.7 ± 0.5	4.5 ± 0.5	*p* = 0.024	*p* < 0.001	*p* < 0.001
eWAT FCV, pL (*n* = 5)	52.5 ± 9.7	113 ± 27.1	*p* = 0.327	296 ± 50.4	247 ± 92.6	*p* = 0.510	*p* < 0.001	*p* = 0.007
eWAT FCN per depot, millions (*n* = 5)	16 ± 5.4	8.1 ± 1.9	*p* = 0.005	4.2 ± 0.5	8.9 ± 2.4	*p* = 0.127	*p* < 0.001	*p* = 0.974
iBAT, g (*n* = 10)	0.1 ± 0.0	0.2 ± 0.1	*p* < 0.001	0.1 ± 0.0	0.4 ± 0.1	*p* < 0.001	*p* = 0.301	*p* < 0.001
iBAT, %BW (*n* = 10)	0.3 ± 0.1	0.8 ± 0.2	*p* < 0.001	0.4 ± 0.1	0.8 ± 0.2	*p* < 0.001	*p* = 0.885	*p* = 0.609
Brown adipocyte area, um^2^ (*n* = 5)	374 ± 79.7	537 ± 89.1	*p* = 0.038	449 ± 83.1	658 ± 90.2	*p* = 0.007	*p* = 0.528	*p* = 0.157
Liver, g (*n* = 10)	0.9 ± 0.1	1.0 ± 0.1	*p* = 0.973	1.1 ± 0.1	1.6 ± 0.3	*p* < 0.001	*p* = 0.505	*p* < 0.001
Liver, %BW (*n* = 10)	3.7 ± 0.3	3.5 ± 0.4	*p* = 0.543	3.3 ± 0.1	3.9 ± 0.5	*p* = 0.002	*p* = 0.059	*p* = 0.044
Gallbladder, g (*n* = 9–10)	0.019 ± 0.006	0.010 ± 0.003	*p* = 0.001	0.019 ± 0.005	0.013 ± 0.004	*p* = 0.009	*p* = 0.989	*p* = 0.611
Food efficiency, g gained/kcal consumed (*n* = 10)	0.005 ± 0.002	0.006 ± 0.002	*p* = 0.919	0.012 ± 0.003	0.017 ± 0.001	*p* < 0.001	*p* < 0.001	*p* < 0.001
iWAT cholesterol, mg/g (*n* = 8–9)	N/A	N/A	N/A	1.61 ± 0.348	2.41 ± 0.555	*p* = 0.003	N/A	N/A

Data are shown as means ± SD. HFD, high-fat diet; WT, Wild Type; LFABP, Liver Fatty Acid-Binding Protein; BW, body weight; iWAT, inguinal white adipose tissue; FCV, fat cell volume; FCN, fat cell number; eWAT, epididymal white adipose tissue; iBAT, interscapular brown adipose tissue; N/A, not available. Adiposity Index was calculated as the ratio of the sum of the excised adipose depots over BW after fasting. Statistical analysis was performed by using two-way ANOVA with Tukey’s *post hoc* comparisons.

**Table 2:** Differential expression of selected gene sets for HALLMARK, REACTOME, and KEGG pathway enrichment analyses in iWAT of *LFABP* null mice relative to WT.

Pathway Name	NES	FDRq Value	Top Genes
**UPREGULATED**			
**HALLMARK**			
HALLMARK_Cholesterol Homeostasis	2.28	0.000	*Nfil*3, *Tnfrsf*12*a*, *Sc*5*d*, *Mvd*, *Hmgcs*1, *Cyp*51*a*1, *Ldlr*, *Idi*1, *Dhcr*7, *Hsd*17*b*7, *Scd*
HALLMARK_TNFa Signaling via NF-κB	1.93	0.000	*Nfil*3, *Sdc*4, *Trib*1, *Serpine*1, *Dnajb*4, *Spsb*1, *Maff*, *Pmepa*1, *Ldlr*, *Ccn*1, *Cdkn*1*a*, *Cxcl*2, *Dusp*1, *Nr*4*a*1
HALLMARK_mTORC1 Signaling	1.86	0.001	*Nfil*3, *Sc*5*d*, *Insig*1, *Hmgcs*1, *Cyp*51*a*1, *Ldlr*, *Ddit*3, *Cdkn*1*a*, *Acsl*3, *Idi*1, *Tes*, *Bcat*1, *Dhcr*7, *Ifrd*1, *Scd*
HALLMARK_Myc Targets V1	1.75	0.003	*Ifrd*1, *Mrps*18*b*, *Apex*1, *Srsf*3, *Pgk*1, *Nhp*2, *Snrpd*2, *Erh*, *Nop*16, *Tra*2*b*
HALLMARK_Hypoxia	1.69	0.005	*Nfil*3, *Sdc*4, *Serpine*1, *Ppp*1*r*3*c*, *Maff*, *Ccn*1, *Ddit*3, *Cdkn*1*a*, *Vhl*, *Tes*, *Pgf*, *Fosl*2, *Dusp*1
**REACTOME**			
REACTOME_Cholesterol Biosynthesis	2.52	0.000	*Sc*5*d*, *Mvd*, *Hmgcs*1, *Cyp*51*a*1, *Msmo*1, *Idi*1, *Dhcr*7, *Hsd*17*b*7, *Fdft*1, *Sqle*
REACTOME_Activation of Gene Expression by SREBF/SREBP	2.26	0.000	*Sc*5*d*, *Mvd*, *Hmgcs*1, *Cyp*51*a*1, *Smarcd*3, *Idi*1, *Dhcr*7, *Fdft*1, *Sqle*, *Fdps*
REACTOME_Regulation of Cholesterol Biosynthesis by SREBP/SREBF	2.18	0.000	*Sc*5*d*, *Insig*1, *Mvd*, *Hmgcs*1, *Cyp*51*a*1, *Smarcd*3, *Idi*1, *Dhcr*7, *Fdft*1, *Sqle*
REACTOME_Mitochondrial Translation	2.16	0.000	*Mrps*18*b*, *mt-Rnr*1, *mt-Rnr*2, *Mrps*6, *Mrpl*15, *Mrpl*57, *Mrpl*33, *Mrpl*20, *Mrpl*55, *Mrpl*46
REACTOME_Mitochondrial Biogenesis	2.01	0.010	*Esrra*, *Smarcd*3, *Prkag*3, *Alas*1, *Prkag*2, *Tmem*11, *Sod*2, *Prkag*1, *Prkab*2, *Cycs*
**KEGG**			
KEGG_Steroid Biosynthesis	2.06	0.001	*Sc*5*d*, *Cyp*51*a*1, *Msmo*1, *Dhcr*7, *Hsd*17*b*7, *Fdft*1, *Sqle*, *Lss*, *Nsdhl*, *Dhcr*24
KEGG_Spliceosome	2.04	0.001	*Hspa*2, *Hspa*1*l*, *Srsf*9, *Srsf*3, *Ppil*1, *Rbm*8*a*, *Hnrnpk*, *Thoc*3, *Snrpd*2, *Srsf*10
KEGG_Terpenoid Backbone Biosynthesis	2.03	0.001	*Mvd*, *Hmgcs*1, *Idi*1, *Fdps*, *Pmvk*, *Mvk*, *Pdss*1
KEGG_Ribosome	1.79	0.018	*Rpl*3*l*, *Rsl*24*d*1, *Mrpl*13, *Rpl*22*l*1, *Rpl*38, *Fau*, *Rps*28, *Rps*27*l*, *Rpl*13*a*, *Rpl*35
KEGG_Proteasome	1.74	0.026	*Psme*3, *Psmb*3, *Psmd*8, *Psmc*4, *Psmc*2, *Psmb*10, *Psma*2, *Psmb*4, *Psmb*5, *Psmd*13
**DOWNREGULATED**			
**HALLMARK**			
HALLMARK_Interferon-alpha Response	−1.83	0.005	*Ifi*44, *Tmem*140, *Trim*14, *Rtp*4, *Lgals*3*bp*, *Procr*, *Batf*2, *Irf*7, *C*1*s*, *Txnip*
HALLMARK_Epithelial Mesenchymal Transition	−1.57	0.063	*Foxc*2, *Postn*, *Col*1*a*2, *Lama*2, *Ecm*1, *Col*6*a*3, *Anpep*, *Mmp*2, *Mmp*3, *Col*11*a*1, *Col*1*a*1, *Looxl*2, *Slit*2, *Dab*2, *Col*3*a*1, *Spp*1
HALLMARK_Complement	−1.48	0.105	*Cfh*, *C*2, *Cp*, *Timp*2, *Mt*3, *Pla*2*g*7, *C*1*r*, *Lipa*, *Itgam*, *Cd*46, *Ang*
HALLMARK_Coagulation	−1.47	0.080	*Cfh*, *C*2, *Mmp*9, *Mmp*2, *Mmp*3, *C*1*r*, *P*2*ry*1, *Cfd*, *Ang*, *Gp*9
HALLMARK_Bile Acid Metabolism	−1.40	0.124	*Abca*6, *Abcg*4, *Abca*9, *Hsd*3*b*7, *Cat*, *Abca*8, *Abca*1, *Ephx*2, *Pex*6, *Retsat*
**REACTOME**			
REACTOME_Collagen Degradation	−2.30	0.000	*Elane*, *Col*1*a*2, *Col*6*a*3, *Col*4*a*5, *Mmp*9, *Mmp*2, *Col*6*a*1, *Mmp*3, *Col*11*a*1, *Col*1*a*1
REACTOME_Assembly of Collagen Fibrils and Other Multimeric Structures	−2.25	0.000	*Col*1*a*2, *Col*6*a*3, *Col*4*a*5, *Mmp*9, *Col*6*a*1, *Mmp*3, *Col*11*a*1, *Col*1*a*1, *Loxl*2, *Col*3*a*1
REACTOME_Initial Triggering of Complement	−2.21	0.000	*C*2, *C*1*qb*, *C*1*r*, *Cfd*, *Cfp*, *C*1*s*, *C*1*qc*, *C*4*b*, *C*1*qa*, *C*3
REACTOME_Collagen Formation	−2.19	0.000	*Col*1*a*2, *Col*6*a*3, *Col*4*a*5, *Mmp*9, *Col*6*a*1, *Mmp*3, *Col*11*a*1, *Col*1*a*1, *Loxl*2, *Col*3*a*1
REACTOME_Collagen Biosynthesis and Modifying Enzymes	−2.16	0.001	*Col*1*a*2, *Col*6*a*3, *Col*4*a*5, *Col*6*a*1, *Col*11*a*1, *Col*1*a*1, *Col*3*a*1, *P*3*h*3, *Col*26*a*1, *Adamts*14
**KEGG**			
KEGG_Renin Angiotensin System	−2.14	0.000	*Agt*, *Agtr*1, *Anpep*, *Ace*, *Enpep*, *Ace*2, *Cpa*3, *Cma*1
KEGG_ECM Receptor Interaction	−2.07	0.000	*Col*1*a*2, *Lama*2, *Col*6*a*3, *Lamb*1, *Col*6*a*1, *Col*11*a*1, *Col*1*a*1, *Col*3*a*1, *Lamc*3, *Spp*1
KEGG_Lysosome	−1.98	0.001	*Slc*11*a*1, *Cd*68, *Lipa*, *Atp*6*v*0*d*2, *Hexa*, *Lgmn*, *Asah*1, *Sort*1, *Ctsk*, *Ctsc*
KEGG_Glycosaminoglycan Degradation	−1.97	0.001	*Hexa*, *Hs*3*st*3*b*1, *Hyal*2, *Hgsnat*, *Hyal*1, *Glb*1, *Gusb*, *Hyal*3, *Gns*, *Idua*
KEGG_Hematopoietic Cell Lineage	−1.95	0.001	*Il*1*r*2, *Gypa*, *Anpep*, *Cd*14, *Il*5, *Fcgr*1*a*, *Epor*, *Itgam*, *Gp*9, *Gp*5

## Data Availability

All data needed to evaluate the conclusions in the paper are present in the paper and/or the Supplementary Materials. Further information and requests for resources and reagents should be directed to the lead contacts J.S. (storch@sebs.rutgers.edu) and A.D. (anastasia.diolintzi@ucsf.edu). RNA-Seq data have been deposited in the GEO (Gene Expression Omnibus database), with the following GEO Series number: GSE277001.

## Supplementary Materials

The following supporting information can be downloaded at: https://www.mdpi.com/article/10.3390/cells14110760/s1, Figure S1: iWAT RNAseq volcano plot; Figure S2: STRING diagram of protein–protein interactions of differentially expressed genes (DEGs); Table S1: Diet compositions of LF and HF diets; Table S2: HALLMARK, KEGG and REACTOME gene sets enriched in the iWAT of HF-fed *LFABP*^−/−^ male mice; Table S3: HALLMARK, KEGG and REACTOME gene sets downregulated in the iWAT of HF-fed *LFABP*^−/−^ male mice.

## Abbreviations

The following abbreviations are used in this manuscript:

LFABP^−/−^	Liver Fatty Acid-Binding Protein Null
HFD	High-fat diet
SAT	Subcutaneous adipose tissue
WT	Wild type
LCFAs	Long-chain fatty acids
MAGs	Monoacylglycerols
eCBs	Endocannabinoids
BAs	Bile acids
MHO	Metabolically healthy obese
FFA	Free fatty acid
AT	Adipose tissue
APCs	Adipocyte progenitor cells
ECM	Extracellular matrix
IR	Insulin resistance
sWAT	Subcutaneous white adipose tissue
vWAT	Visceral WAT
BAT	Brown adipose tissue
DIO	Diet-induced obesity
LFD	Low-fat diet
MRI	Magnetic Resonance Imaging
H&E	Hematoxylin and eosin
CLSs	Crown-like structures
FCV	Fat cell volume
FCN	Fat cell number
TAG	Triacylglycerol
iWAT	Inguinal WAT
eWAT	Epididymal WAT
iBAT	Interscapular BAT
GSEA	Gene set enrichment analysis
MSigDB	Metabolic Signatures Database
NES	Normalized enrichment score
FDR	False discovery rate
RPKM	Reads Per Kilobase per Million mapped
DEGs	Differentially expressed genes
PBS	Phosphate-buffered saline
BW	Body weight
CYP2E1	Cytochrome P450 family 2 subfamily E member 1
Areg	Adipogenesis regulator
FAM13A	Family with sequence similarity 13 member A
Hr	Hairless
ESRRA	Estrogen-Related Receptor α
DUSP1	Dual-Specificity Phosphatase 1
Cdkn1a	Cyclin-Dependent Kinase Inhibitor 1A
Gadd45g	Growth Arrest and DNA-Damage-Inducible 45 Gamma
Postn	Periostin
Hmgcs1	3-hydroxy-3-methylglutaryl-CoA synthase 1
mTORC1	Mechanistic Target of Rapamycin Complex 1
Scd2	Stearoyl-CoA-Desaturase 2
Tnfrsf12a	Tumor Necrosis Factor Receptor Superfamily Member 12A
TWEAK	TNF-like weak inducer of apoptosis
Col	Collagen
Lama2	Laminin α-2 chain
Spp1	Osteopontin
Ecm1	Extracellular Matrix Protein 1
Thbs1	Thrombospondin-1
MMP	Matrix Metalloproteinase
TIMP2	Tissue Metalloproteinase Inhibitor 2
Serpine1	Serine Protease Inhibitor Clade E Member 1
PAI-1	Plasminogen Activator Inhibitor-1
KO	Knockout
Sdc4	Syndecan 4
ASPCs	Adipose stem and progenitor cells
MUFAs	Monounsaturated fatty acids
PLs	Phospholipids
LPS	Lipopolysaccharide
TNFRSFs	TNFR Superfamily Members
MSCs	Mesenchymal Stem Cells
AEA	Anandamide
Chol	Cholesterol
