# Supplementary material for: Novel Hyperplastic Expansion of White Adipose Tissue Underlies the Metabolically Healthy Obese Phenotype of Male LFABP Null Mice
Source: Cells. 2025 May 22;14(11):760. doi: 10.3390/cells14110760 (PMC12153764; doi:10.3390/cells14110760)
Supplement: Supplementary file 1 [file cells-14-00760-s001.zip › cells-3601422-supplementary.pdf]

Supplementary Materials for

Novel hyperplastic expansion of white adipose tissue underlies the metabolically healthy obese phenotype of male LFABP null mice

Anastasia Diolintzi *et al.*

*Corresponding author. Email: anastasia.diolintzi@ucsf.edu, storch@sebs.rutgers.edu

This PDF file includes:

Figures S1 to S2
Tables S1 to S3

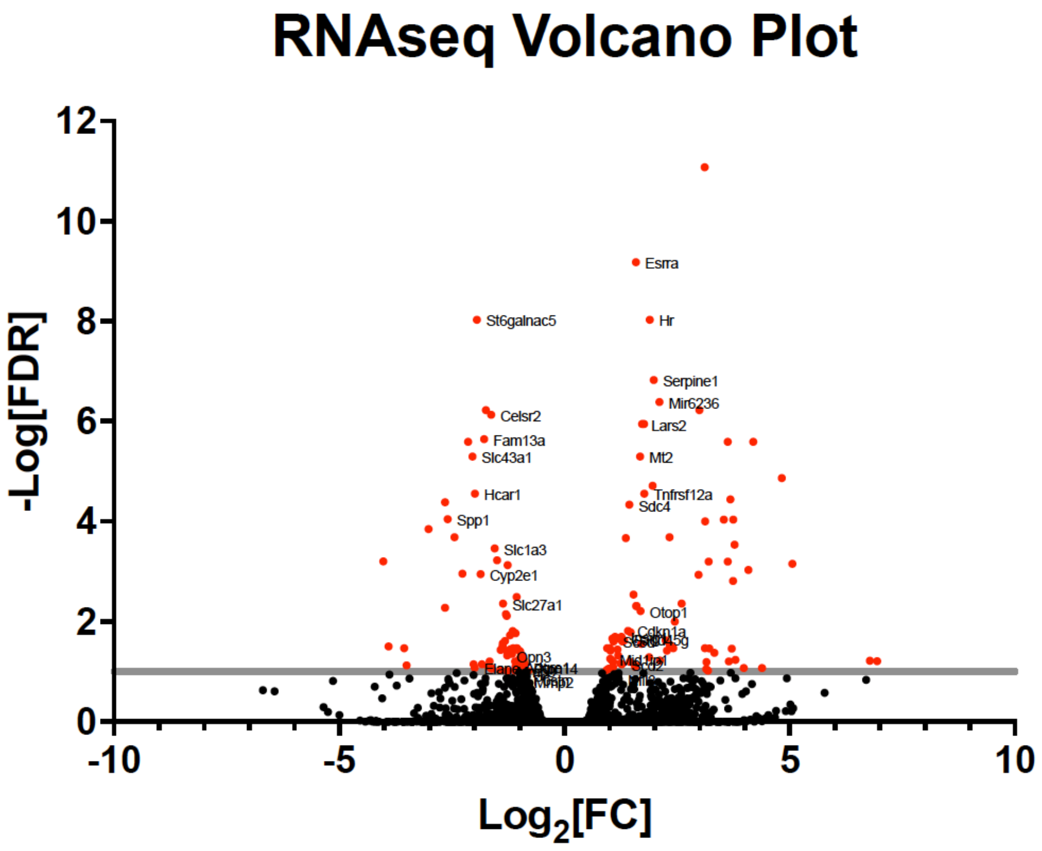

Figure S1. iWAT RNAseq Volcano Plot. Volcano plot of RNAseq analysis for iWAT between LFABP null and WT mice (n = 5 / genotype). The X axis shows the logarithmic value with base 2 for transcript fold change (Log_2[FC]). The Y axis shows the negative logarithmic value of FDRq-values (-Log[FDR]). The solid gray line indicates the -Log[FDR] for FDRq-value < 0.1, *i.e.,* 1. Selected transcript names are shown for some data points.

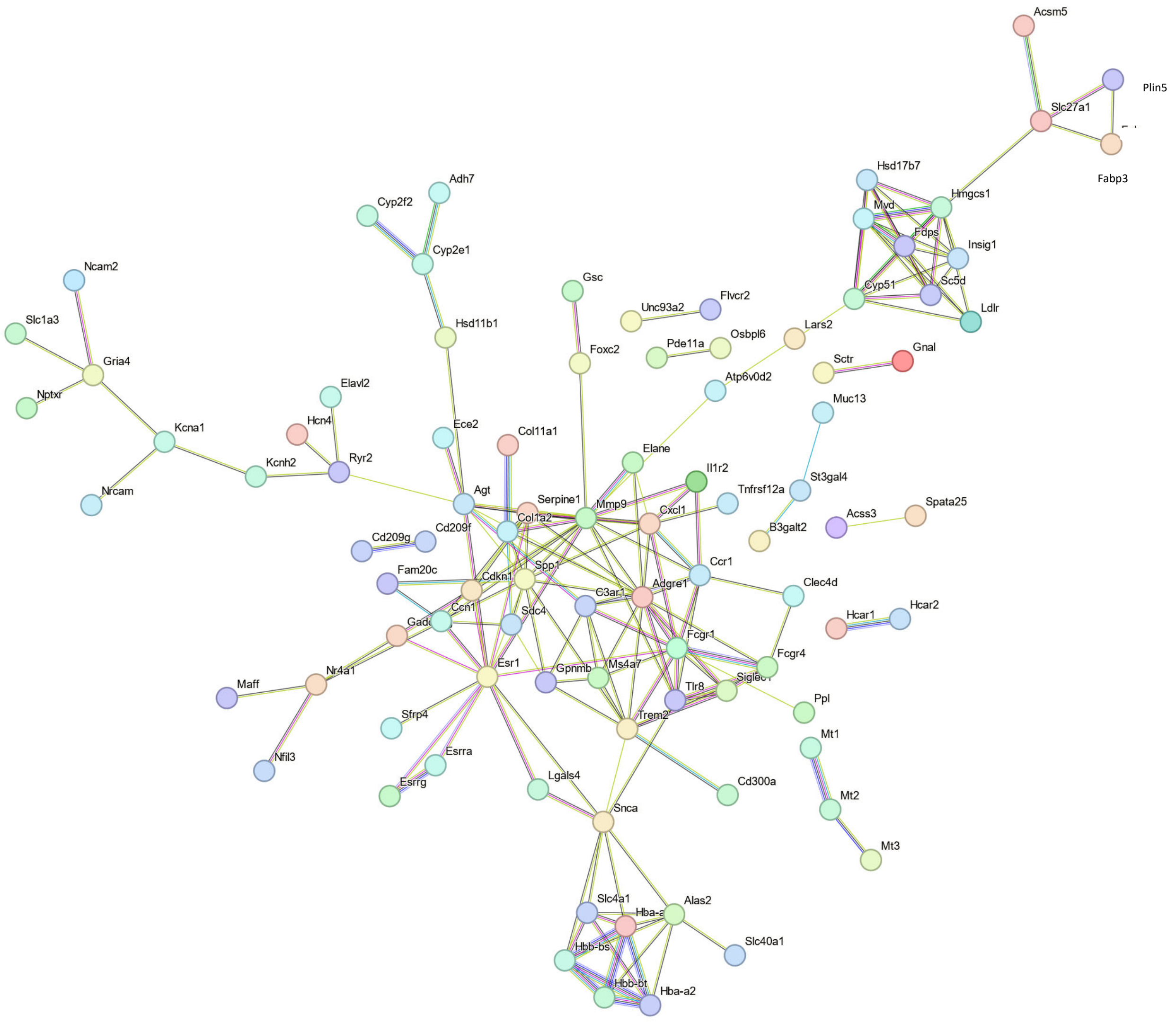

Figure S2. STRING diagram of protein-protein interactions of differentially expressed genes (DEGs). Differentially expressed genes (DEGs) were identified from RNA-seq data using DESeq2 [1] with thresholds of |log2 fold change| > 1 and FDR < 0.1. The protein-coding DEGs retained was submitted to the STRING database (version 12.0) [2]. Potential protein-protein interactions reveal active nodes between protein-coding genes involved with cholesterol biosynthesis, extracellular matrix (ECM) remodeling, and NF-κB signaling.

Table S1. Diet compositions of LF and HF diets. Adjusted from [3]

	LFD			HFD		
	g	*Kcal*	*% Kcal*	*g*	*Kcal*	*% Kcal*
Casein	200	800	19.7	200	800	19.7
L-Cystine	3	12	0.3	3	12	0.3
Corn starch	315	1260	31	72.8	291	7.2
Maltodextrin	35	140	3.5	100	400	9.9
Sucrose	350	1400	34.5	172.8	691	17
Cellulose	50	0	0	50	0	0
Soybean oil	10	90	2.2	10	90	2.2
Lard	8.5	77	1.9	0	0	0
Cocoa butter	26.5	239	5.9	192.5	1733	42.7
High oleic safflower oil	0	0	0	0	0	0
Mineral mix, S10026	10	0	0	10	0	0
Dicalcium phosphate	13	0	0	13	0	0
Calcium carbonate	5.5	0	0	5.5	0	0
Potassium citrate, 1 H_2O	16.5	0	0	16.5	0	0
Vitamin mix, V10001	10	40	1	10	40	1
Choline bitartrate	2	0	0	2	0	0
FD&C yellow dye no. 5	0.05	0	0	0	0	0
FD&C yellow dye no. 40	0	0	0	0.05	0	0
FD&C yellow dye no. 1	0	0	0	0	0	0
Total	1055.05	4057	100	858.15	4057	100

Table S2. HALLMARK, KEGG and REACTOME Gene Sets Enriched in the iWAT of HF-fed LFABP-/- male mice. FDR < 0.1, |FC| > 1.2

**Gene Set**	**NES**	**FDR**
REACTOME_Cholesterol Biosynthesis	2.52	0.000
HALLMARK_Cholesterol Homeostasis	2.28	0.000
REACTOME_Activation of Gene Expression by SREBF/SREBP	2.26	0.000
REACTOME_Regulation of Cholesterol Biosynthesis by SREBP/SREBF	2.18	0.000
REACTOME_Mitochondrial Translation	2.16	0.000
KEGG_Steroid Biosynthesis	2.06	0.001
KEGG_Spliceosome	2.04	0.001
KEGG_Terpenoid Backbone Biosynthesis	2.03	0.001
REACTOME_Mitochondrial Biogenesis	2.01	0.010
REACTOME_Negative Regulation of MAPK Pathway	1.96	0.018
HALLMARK_TNFa Signaling via NFkB	1.93	0.000
REACTOME_Transcriptional Activation of Mitochondrial Biogenesis	1.91	0.028
HALLMARK_mTORC1 Signaling	1.86	0.001
REACTOME_Respiratory Electron Transport	1.85	0.051
REACTOME_Negative Regulation of NOTCH4 Signaling	1.81	0.056
KEGG_Ribosome	1.79	0.018
REACTOME_Selective Autophagy	1.79	0.062
REACTOME_RAF-Independent MAPK1/3 Activation	1.78	0.061
REACTOME_ATF4 Activates Genes in Response to Endoplasmic Reticulum Stress	1.78	0.060
REACTOME_CTLA4 Inhibitory Signaling	1.77	0.067
REACTOME_RAF Activation	1.77	0.065
REACTOME_Signaling by the B Cell Receptor BCR	1.76	0.066
REACTOME_Butyrate Response Factor 1/BRF1 Binds and Destabilizes mRNA	1.76	0.065
HALLMARK_Myc Targets V1	1.75	0.003
REACTOME_Regulation of Gene Expression in Beta Cells	1.74	0.071
KEGG_Proteasome	1.74	0.026
KEGG_RNA Degradation	1.70	0.030
KEGG_SNARE Interactions in Vesicular Transport	1.70	0.030
REACTOME_Regulation of RUNX3 Expression and Activity	1.70	0.083
HALLMARK_Hypoxia	1.69	0.005
REACTOME_SHC-Mediated Cascade FGFR4	1.69	0.085
REACTOME_Energy-Dependent Regulation of mTOR by LKB1 AMPK	1.68	0.090
REACTOME_Downstream Signaling Events of B Cell Receptor/BCR	1.67	0.089

REACTOME_Degradation of Beta Catenin by the Destruction Complex	1.67	0.089
REACTOME_TNFR2 Non-Canonical NF-kB Pathway	1.67	0.090
REACTOME_Signaling by NOTCH4	1.67	0.088
REACTOME_The Role of GTSE1 in G2/M Progression After G2 Checkpoint	1.66	0.091
KEGG_Long-Term Depression	1.64	0.046
REACTOME_PI3K Cascade FGFR4	1.64	0.096
REACTOME_TP53 Regulates Metabolic Genes	1.64	0.095
HALLMARK_Androgen Response	1.63	0.008
KEGG_Huntington's Disease	1.63	0.049
KEGG_Cardiac Muscle Contraction	1.60	0.063
REACTOME_Dectin1 Mediated Noncanonical NF-kB Signaling	1.60	0.098
HALLMARK_Oxidative Phosphorylation	1.58	0.010
HALLMARK_Myc Targets V2	1.57	0.010
KEGG_MAPK Signaling Pathway	1.57	0.073
KEGG_RNA Polymerase	1.53	0.094
HALLMARK_p53 Pathway	1.48	0.025
HALLMARK_PI3k/Akt/mTOR Signaling	1.33	0.080
HALLMARK_Estrogen Response Early	1.31	0.089

Table S3. HALLMARK, KEGG and REACTOME Gene Sets Downregulated in the iWAT of HF-fed LFABP-/- male mice. FDR < 0.1, |FC| > 1.2

Gene Set	NES	FDR
REACTOME_Collagen Degradation	2.30	0.000
REACTOME_Binding and Uptake of Ligands by Scavenger Receptors	2.30	0.000
REACTOME_Assembly of Collagen Fibrils and Other Multimeric Structures	2.25	0.000
REACTOME_Initial Triggering of Complement	2.21	0.000
REACTOME_Collagen Formation	2.19	0.000
REACTOME_Collagen Biosynthesis and Modifying Enzymes	2.16	0.001
REACTOME_Degradation of the Extracellular Matrix	2.16	0.000
REACTOME_Collagen Chain Trimerization	2.15	0.001
KEGG_Renin Angiotensin System	2.14	0.000
REACTOME_Scavenging by Class A Receptors	2.11	0.001
REACTOME_Complement Cascade	2.09	0.001
REACTOME_Activation of Matrix Metalloproteinases	2.08	0.001
REACTOME_MET Activates PTK2 Signaling	2.07	0.002
KEGG_ECM Receptor Interaction	2.07	0.000
REACTOME_Branched-Chain Amino Acid Metabolism	2.04	0.002
REACTOME_Metabolism of Angiotensinogen to Angiotensins	2.00	0.004
REACTOME_MET Promotes Cell Motility	1.97	0.005
KEGG_Lysosome	1.98	0.001
KEGG_Glycosaminoglycan Degradation	1.97	0.001
REACTOME_ECM Proteoglycans	1.96	0.005
KEGG_Hematopoietic Cell Lineage	1.95	0.001
REACTOME_Immunoregulatory Interactions Between a Lymphoid and a Non-Lymphoid Cell	1.93	0.007
REACTOME_Crosslinking of Collagen Fibrils	1.93	0.007
REACTOME_Extracellular Matrix Organization	1.92	0.007
REACTOME_Antimicrobial Peptides	1.88	0.011
REACTOME_Laminin Interactions	1.86	0.014
HALLMARK_Interferon-alpha Response	1.83	0.005
REACTOME_Integrin Cell Surface Interactions	1.78	0.030
REACTOME_Xenobiotics	1.75	0.041
REACTOME_FCGR3A-Mediated IL10 Synthesis	1.74	0.045
REACTOME_Anchoring Fibril Formation	1.71	0.059
REACTOME_Interleukin-10 Signaling	1.70	0.061
KEGG_ABC Transporters	1.70	0.014
KEGG_Propanoate Metabolism	1.69	0.015
REACTOME_O-Linked Glycosylation	1.68	0.073

REACTOME_Glycosphingolipid Metabolism	1.65	0.097
REACTOME_ABC Transporters in Lipid Homeostasis	1.64	0.096
REACTOME_Sialic Acid Metabolism	1.64	0.099
KEGG_Complement and Coagulation Cascades	1.60	0.030
HALLMARK_Epithelial Mesenchymal Transition	1.57	0.063
KEGG_Sphingolipid Metabolism	1.53	0.048
KEGG_Valine, Leucine and Isoleucine Degradation	1.52	0.050
KEGG_Other Glycan Degradation	1.47	0.064
HALLMARK_Coagulation	1.47	0.080
KEGG_Metabolism of Xenobiotics by Cytochrome P450	1.43	0.085
KEGG_O-Glycan Biosynthesis	1.41	0.098
HALLMARK_Xenobiotic Metabolism	1.39	0.099

1. Love, M. I., W. Huber and S. Anders. "Moderated estimation of fold change and dispersion for rna-seq data with deseq2." *Genome Biol* 15 (2014): 550. 10.1186/s13059-014-0550-8. https://www.ncbi.nlm.nih.gov/pubmed/25516281.
2. Szklarczyk, D., R. Kirsch, M. Koutrouli, K. Nastou, F. Mehryary, R. Hachilif, A. L. Gable, T. Fang, N. T. Doncheva, S. Pyysalo*, et al.* "The string database in 2023: Protein-protein association networks and functional enrichment analyses for any sequenced genome of interest." *Nucleic Acids Res* 51 (2023): D638-D46. 10.1093/nar/gkac1000. https://www.ncbi.nlm.nih.gov/pubmed/36370105.
3. Gajda, A. M., Y. X. Zhou, L. B. Agellon, S. K. Fried, S. Kodukula, W. Fortson, K. Patel and J. Storch. "Direct comparison of mice null for liver or intestinal fatty acid-binding proteins reveals highly divergent phenotypic responses to high fat feeding." 288 (2013): 30330-44. 10.1074/jbc.m113.501676. https://dx.doi.org/10.1074/jbc.m113.501676.
